# Aging aggravated liver ischemia and reperfusion injury by promoting oxidized mtDNA mediated-macrophage pyroptosis through acetylated MCU-dependent calcium uptake

**DOI:** 10.1038/s41420-025-02746-9

**Published:** 2025-10-07

**Authors:** Xin-Yi Wu, Rui Wang, Qi Zhang, Tao Liu, Jun-Yan Liu, Xue-Song Xu, Jun-Hua Gong

**Affiliations:** 1 Department of Hepatobiliary Surgery, The Second Affiliated Hospital of Chongqing Medical University, Chongqing, China; 2 Department of Rehabilitation, The Second Affiliated Hospital of Chongqing Medical University, Chongqing, China

**Keywords:** Immune cell death, Inflammatory diseases

## Abstract

The shortage of liver donors for liver transplantation is currently an urgent problem. Elderly donors have become an important source of donor livers, but they are more prone to ischemia reperfusion injury (IRI) in liver transplantation. Therefore, exploring the effects and mechanisms of aging on liver IRI will provide a new theoretical basis for improving the survival rate of liver transplant patients. We constructed a mouse model of liver ischemia for 90 min and reperfusion for 6 or 24 h, and found that compared with young liver, the recovery of liver function in aged liver after IRI was slower. Detection of macrophage pyroptosis revealed that it was an important factor for aging deferring liver function restoration. Mechanistically, we demonstrated that aging triggered mitochondrial permeability transition pore (mPTP) channel opening to promote the release of Oxidized mtDNA (Ox-mtDNA), thereby inducing macrophage pyroptosis. Moreover, the activity of mPTP channel was mainly dependent on calcium uptake by acetylated mitochondrial calcium uniporter (MCU). These results illustrated that cytoplasmic Ox-mtDNA-induced macrophage pyroptosis was a key factor for aging exacerbating liver IRI. Calcium uptake via acetylated MCU triggered mPTP channel opening, which is an important mechanism for Ox-mtDNA release from mitochondria into the cytoplasm.

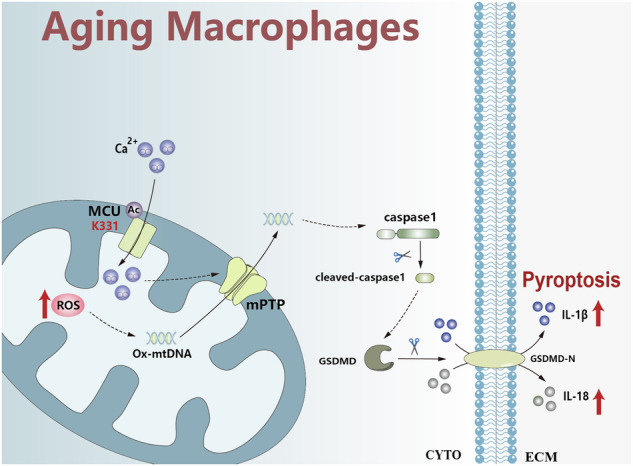

## Introduction

Liver transplantation is currently recognized as the only effective treatment for end-stage liver disease and the number of patients awaiting transplantation increases rapidly [1]. However, the availability of liver donors has become insufficient [2]. Thus, expanding the availability of donor livers is crucial for addressing the shortage of suitable liver in clinical liver transplantation. With the increase in the elderly population, elderly (>60 years old) donors have become an important source of donor livers, but many studies have been reported that elderly donor grafts are more prone to ischemia-reperfusion injury (IRI) [3] and have lower long-term survival [4–6]. Hence, it is crucial to study the reasons why aging exacerbates liver IRI and optimize the use of elderly liver transplantation.

It has been proposed that warm liver IRI has two distinct phases [7]. Compared with ischemia injury, innate-immune-dominated tissue inflammatory response initiated by liver macrophages after reperfusion involves more cytotoxic mechanisms [8]. Pyroptosis is a novel type of programmed cell death that relies on inflammatory caspases (mainly caspase-1, 4, 5, 11) to cleave full-length gasdermin D (GSDMD) to GSDMD-N, accompanied by the release of a large number of pro-inflammatory factors such as interleukin(IL)-1β and IL-18 [9, 10]. According to a previous study [11], caspase1-GSDMD processing mainly occurred in macrophages, but not in hepatocytes, and induced the inflammatory response during liver IRI. Importantly, recent studies have shown that aging promoted macrophage pyroptosis in other disease [12, 13]. However, it is currently unclear whether macrophage pyroptosis is the important mechanism by which aging aggravates liver IRI.

In aging-related degenerative disorders, mtDNA undergoes oxidative damage (Oxidized mtDNA, Ox-mtDNA) and promotes inflammation via the mitochondrial permeability transition pore (mPTP) channel opening [14–16]. The mPTP channel is defined as a nonselective, voltage-dependent pore within the inner membrane of mitochondria that allows for the passive diffusion of solutes up to 1.5 kDa in size [17]. Importantly, recent study indicated that intracellular calcium (Ca^2+^) overload, but not reactive oxygen species (ROS), induce the opening of the mPTP channel [18]. However, the mechanism by which Ca^2+^ affects the mPTP channel in aging-induced macrophages during liver IRI has not been fully elucidated.

Lysine acetylation is a crucial post-translational modification that regulates a wide range of molecular processes in mammalian cells [19]. Interestingly, Aged liver is closely related to high levels of acetylation [20]. Moreover, accumulated studies reported that protein acetylation in mitochondria has taken center stage, revealing that 63% of mitochondrially localized proteins contain lysine acetylation sites [19, 21]. Consequently, we speculated that aging may trigger the mPTP channel opening by inducing mitochondrial MCU acetylation to uptake Ca^2+^. But the above hypothesis still needs further confirmation.

In this study, we found that aging delayed liver recovery after IRI, and this phenomenon was caused by macrophage pyroptosis which was triggered by the mPTP channel-mediated Ox-mtDNA release. Mechanistically, we further elucidated that the opening of the mPTP channel was primarily due to mitochondrial Ca^2+^ uptake, which was facilitated by acetylated MCU.

## Results

### Aging delayed the recovery of liver function after IRI

**Fig. 1:**
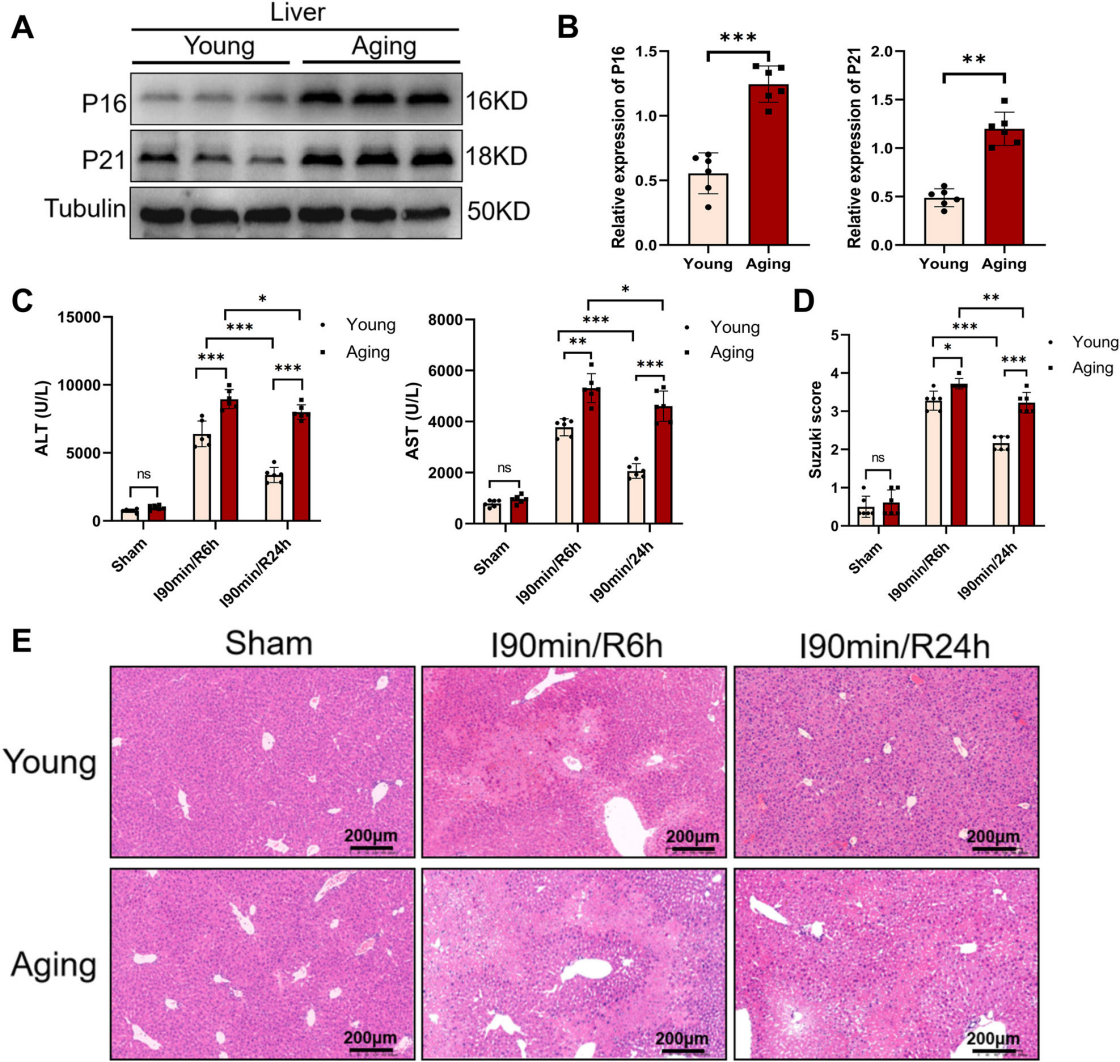
Aging delayed the recovery of liver function after IRI. **A**, **B** The levels of P16 and P21 in young and aged mice were measured by WB. Liver of young and aged mice were subjected to 90 min of warm ischemia followed by 6 or 24 h of reperfusion, respectively. **C** Serum levels of ALT and AST were measured. **D**, **E** average Suzuki scores were based on H&E-stained liver sections from different groups of mice (scale bar, 200 μm). All data are shown as the mean ± SD (*n* = 6). ***p < 0.001, **p < 0.01 and *p < 0.05.

Firstly, we detected the aging markers P16 and P21 in the livers of young and aged mice using WB. Compared with the young group, the expression of P16 and P21 was increased in the aging group (Fig. 1A, B). Then, to determine the effect of aging on liver IRI, liver of young and aged mice were subjected to IRI or sham procedure. Under sham surgery, there was no significant difference in ALT and AST levels between the young and aging groups. After 90 min of liver ischemia and 6 h of reperfusion, the levels of ALT and AST in both groups significantly increased. But interestingly, after 24 h of reperfusion, the level of ALT and AST in the young group decreased more significantly than those in the aging group (Fig. 1C). The H&E staining results also showed that there was no significant liver injury in the young and aging groups with sham surgery. For the young group, sinusoidal congestion, hepatocellular edema, vacuolization and necrosis in liver tissue were exhibited after 6 h of reperfusion but improved after 24 h. However, for the aging group, the above liver injury phenotype was more serious after 6 h of reperfusion but no significant functional recovery after 24 h (Fig. 1D, E). These results indicated that aging delayed the recovery of liver function after IRI.

### Aging aggravated macrophage pyroptosis in liver IRI

**Fig. 2:**
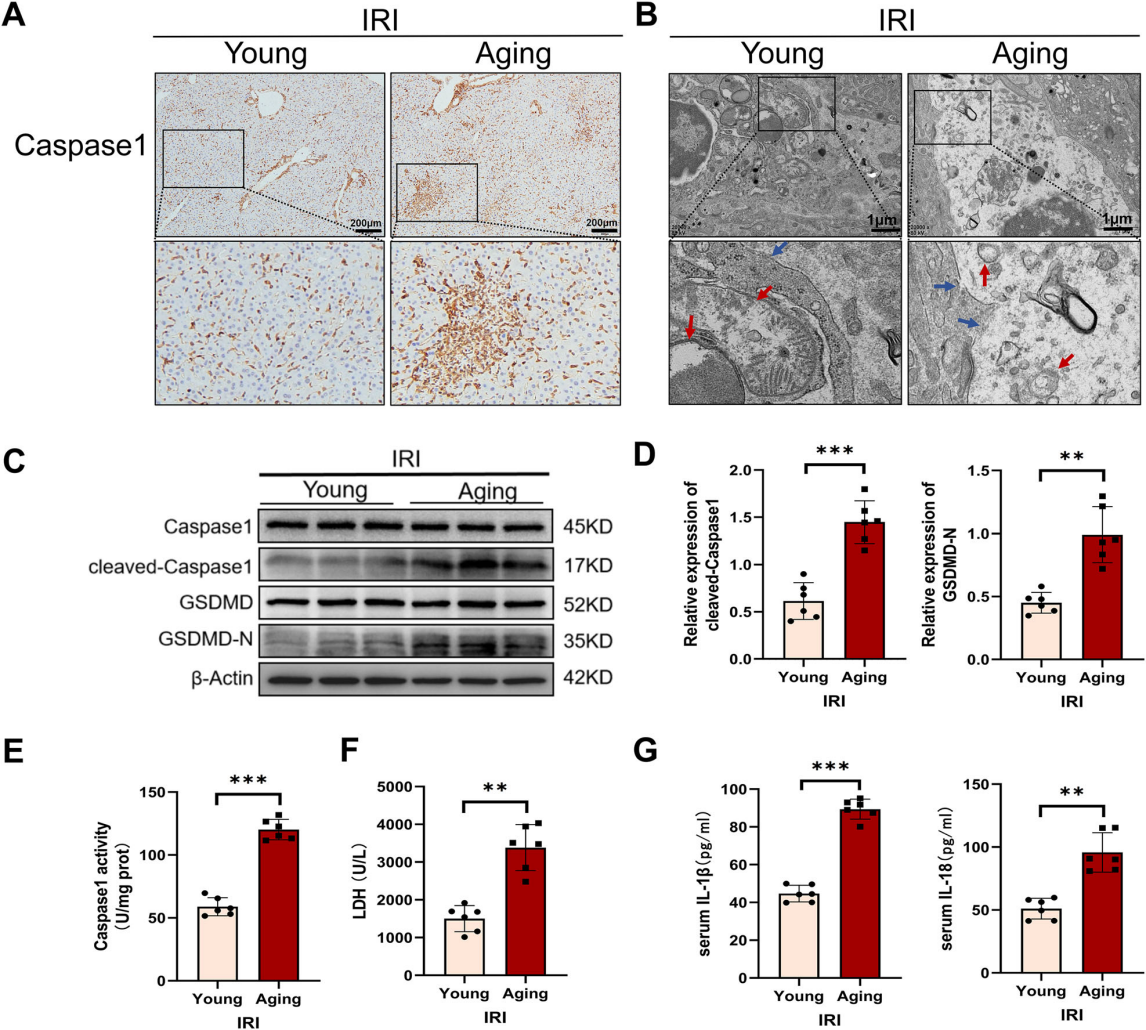
Aging aggravated macrophage pyroptosis in liver IRI. Liver in young and aged mice were subjected to ischemia for 90 min followed by reperfusion for 24 h. **A** Caspase1 was detected using IHC in liver tissue sections (scale bar, 200 μm). **B** TEM was used to observe the ultrastructural changes in macrophages (magnification, 20,000×). The red arrow indicates the incomplete structure of an organelle, and the blue arrow indicates discontinuity in the cell membrane. **C**, **D** Liver macrophages in each group were isolated, and the levels of Caspase1, Cleaved-Caspase1, GSDMD and GSDMD-N were measured by WB. **E** Liver macrophages in each group were isolated, and caspase1 activity was determined with the caspase1 assay kit. **F** Serum levels of LDH were measured. **G** The levels of serum inflammatory factors (IL-1β and IL-18) were tested by ELISA. All data are shown as the mean ± SD (*n* = 6). ***p < 0.001, **p < 0.01 and *p < 0.05.

Aging is associated with an increase in the levels of inflammatory cytokines in the liver and induces macrophage pyroptosis [22, 23]. Pyroptosis, a proinflammatory form of programmed cell death, in innate immune cells aggravates liver IRI and aging disease [13, 24]. Given that, we evaluated whether the delayed recovery of liver IRI due to aging was related to macrophage pyroptosis. IHC showed that after 24 h of reperfusion, a significant increase expression of caspase1 in nonparenchymal cells in the aging group (Fig. 2A). TEM further observed that the membrane discontinuity, cytoplasmic edema, and organelle incompleteness of macrophages in the aging group were more severe than those in the young group (Fig. 2B). Furthermore, we used our TEM analysis protocol to quantify mitochondrial cristae changes, and found that the cristae score was reduced in the aging group relative to the young group (Supplementary Fig. S4A). As shown in Fig. 2C, D, Liver macrophages in each group were isolated, and the expression level of cleaved-caspase1 and GSDMD-N in the aging group was more than those in the young group. Furthermore, the caspase-1 activity in macrophages of the aging group was markedly elevated compared to that of the young group (Fig. 2E). Aging further promoted the secretion and release of LDH, IL-1β and IL-18 in the liver (Fig. 2F, G). The above results showed that aging promoted macrophage pyroptosis in liver IRI, suggesting that macrophage pyroptosis was an important mechanism for aging deferring liver function restoration.

### Aging promoted the release of numerous Ox-mtDNA from mitochondria into the cytoplasm of macrophages

**Fig. 3:**
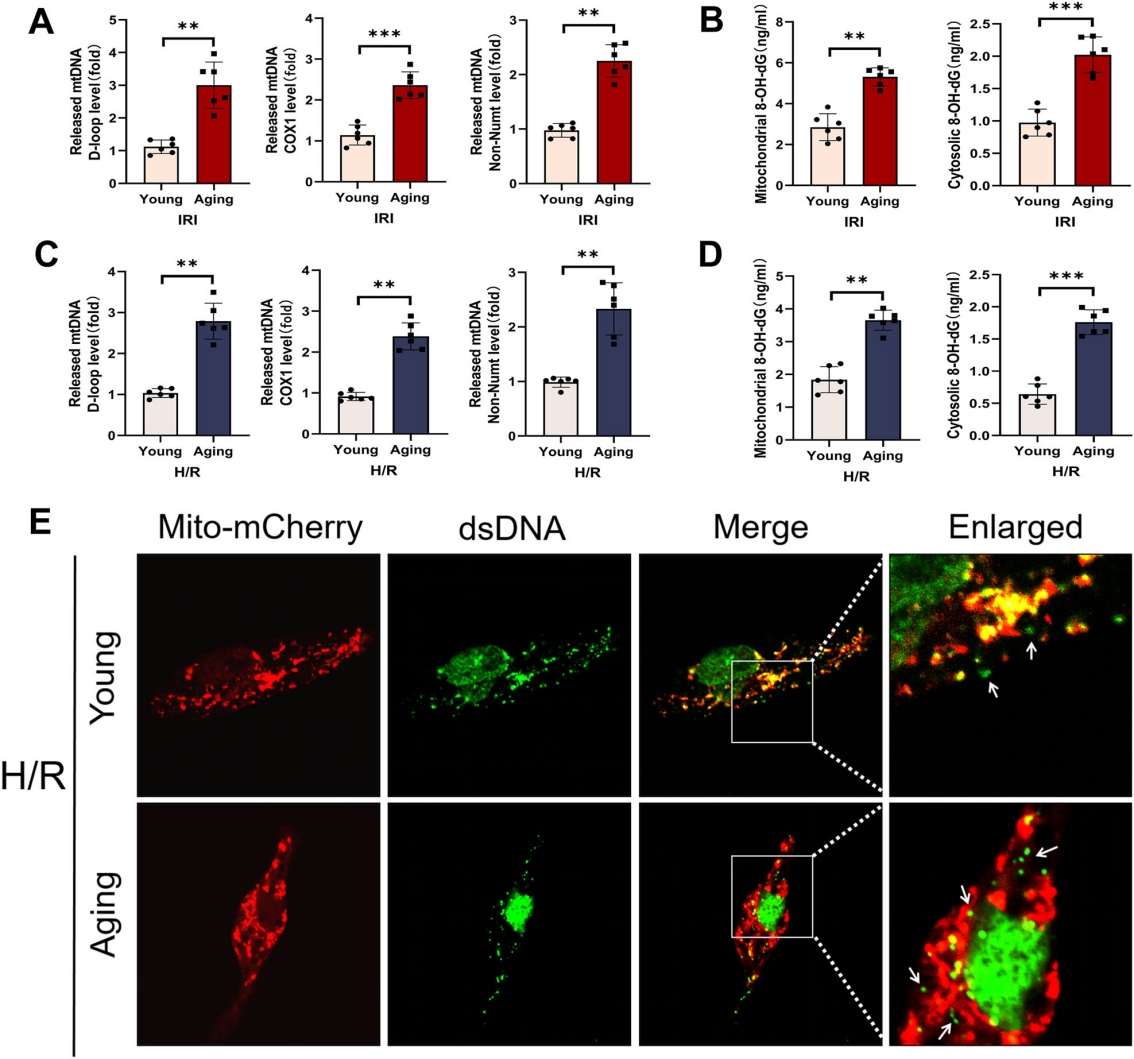
Increased Ox-mtDNA in the cytoplasm of aging-induced macrophages. Young and aged mice were subjected to ischemia for 90 min followed by reperfusion for 24 h. Liver macrophages in each group were isolated. **A** Relative cytosolic mtDNA amounts in each group. The relative ratios of D-loop mtDNA, Cox1 mtDNA, or Non-NUMT mtDNA are tested by qPCR. **B** 8-OH-dG from the mitochondrial (left) or cytosol (right) were quantified using 8-OH-dG ELISA Kit. RAW264.7 cells were co-cultured with the supernatant from normal or aged AML12 cells for 24 h, followed by treatment with H/R. **C** Relative cytosolic mtDNA amounts in each group. The relative ratios of D-loop mtDNA, Cox1 mtDNA, or Non-NUMT mtDNA are tested by qPCR. **D** 8-OH-dG from the mitochondrial (left) or cytosol (right) were quantified by ELISA. **E** The colocalization of Mito-mCherry (red) and dsDNA (green) was detected by confocal microscopy. All data are shown as the mean ± SD (*n* = 6). ***p < 0.001, **p < 0.01 and *p < 0.05.

Research studies have reported that due to exposure to high levels of ROS, mtDNA was more susceptible to oxidative damage than nuclear DNA [25]. Ox-mtDNA leaked into the cytosol and stimulated caspase1-mediated inflammation in aged macrophages [26, 27]. Therefore, to verify whether aging promoted Ox-mtDNA release of macrophages, liver macrophages were isolated from the young and aging groups after 24 h of reperfusion. Mitochondria were isolated and detected for β-actin and Cox4 by WB (Supplementary Fig. S1A). As shown in Fig. 3A, B, compared with the young group, cytoplasmic and mitochondria 8-OH-dG (DNA oxidative damage marker) of liver macrophages in the aging group were both significantly increased, suggesting mtDNA undergone oxidative damage. The aging group had higher mtDNA D-loop, Cox1 and Non-NUMT levels in the cytoplasm of liver macrophages, indicating an increase in cytoplasmic mt-DNA.

In vitro, we induced aging of AML12 cells with etoposide and detected the expression of P16 and P21 in AML12 cells using WB. The expression of P16 and P21 increased in AML12 cells induced by etoposide (Supplementary Fig. S1B, C). Then, RAW264.7 cells were co-cultured with the supernatant from normal or aged AML12 cells, followed by treatment with H/R (Supplementary Fig. S1D). Mitochondria were isolated and detected by WB (Supplementary Fig. S1E). As shown in Fig. 3C, D, cytoplasmic and mitochondria 8-OH-dG in the aging group were both remarkably increased. Meanwhile, there are more mtDNA in the cytoplasm in the aging group. The confocal images showed that compared with the young group, more absence of co-localization between dsDNA and Mito-mCherry in the aging group (Fig. 3E).

The above results suggested that aging promoted oxidative damage to mtDNA and releases it from mitochondria into the cytoplasm of macrophages.

### Aging induced macrophage pyroptosis by promoting mPTP channel mediated Ox-mtDNA release

**Fig. 4:**
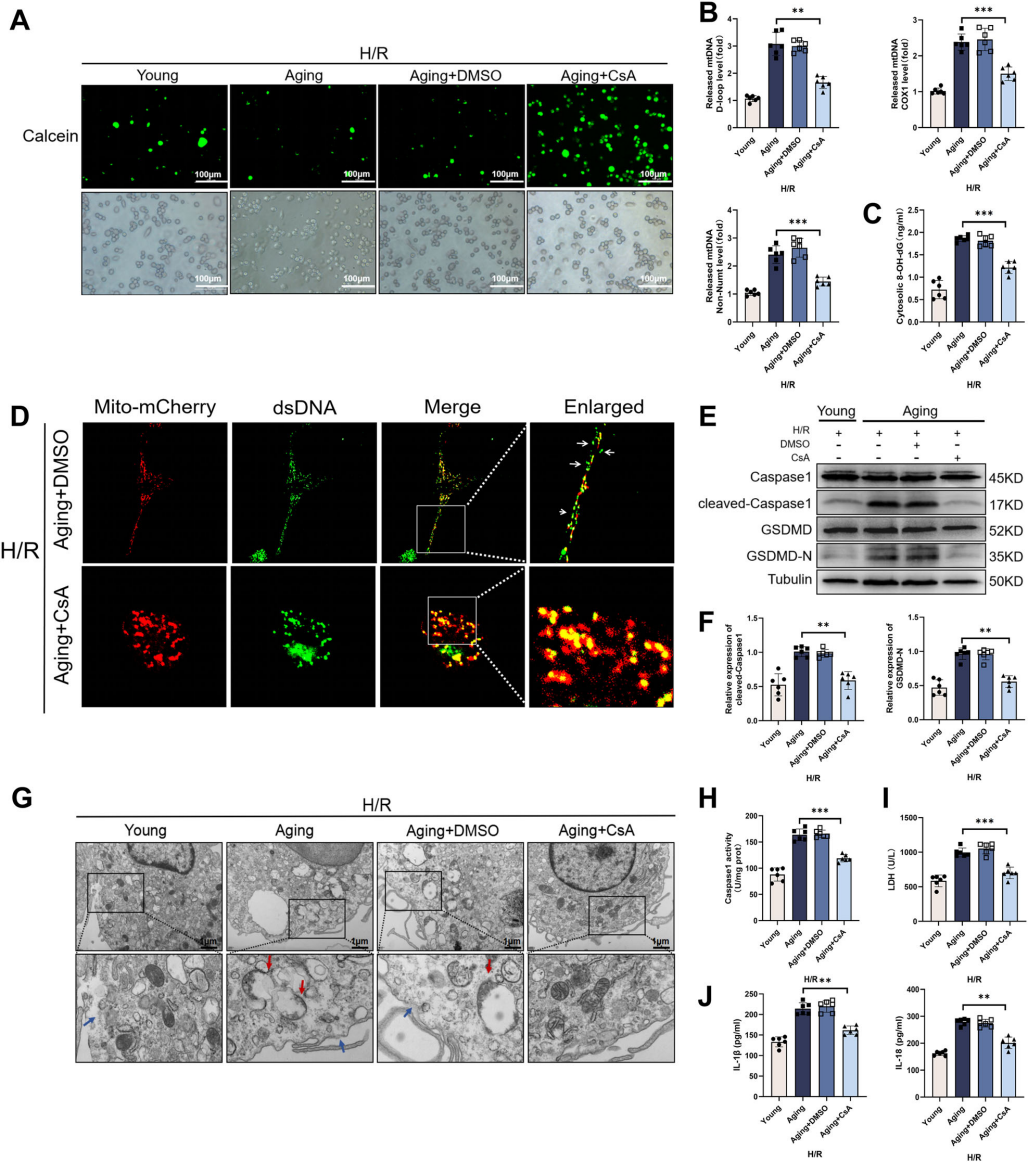
Inhibition of mPTP channel-mediated Ox-mtDNA release alleviated aging-induced macrophage pyroptosis during H/R. RAW264.7 cells were co-cultured with the supernatant from normal or aged AML12 cells in the absence or presence of CsA (1 μM) for 24 h followed by treatment with H/R. **A** Representative of immunofluorescence staining of Calcein (green) and corresponding cell density (white light). **B** Relative cytosolic mtDNA amounts in each group. The relative ratios of D-loop mtDNA, Cox1 mtDNA, or Non-NUMT mtDNA are tested by qPCR. **C** 8-OH-dG in cytosol were quantified using ELISA. **D** The colocalization of Mito-mCherry (red) and dsDNA (green) was detected by confocal microscopy. **E**, **F** The levels of Caspase1, Cleaved-Caspase1, GSDMD and GSDMD-N were measured by WB. **G** TEM was used to observe the ultrastructural changes in macrophages (magnification, 20,000×). The red arrow indicates the incomplete structure of an organelle, and the blue arrow indicates discontinuity in the cell membrane. **H** Caspase1 activity was determined with the caspase1 assay kit. **I** Supernatant levels of LDH were measured by LDH assay kit. **J** Supernatant levels of IL-1β and IL-18 were tested by ELISA. All data are shown as the mean ± SD (*n* = 6). ***p < 0.001, **p < 0.01 and *p < 0.05.

The mPTP channel is located on the mitochondrial membrane and a key channel for Ox-mtDNA release from mitochondrial to the cytoplasm to activate inflammation [16]. However, it is currently unclear whether aging exacerbates macrophage pyroptosis by promoting Ox-mtDNA release. Here, we used the mPTP channel inhibitor cyclosporine A (CsA) that inhibiting mPTP channel opening. In RAW264.7 cells induced by aging microenvironment during H/R, calcein fluorescence decreased, suggesting an increase in open mPTP channels, but CsA blocked the decrease of calcein fluorescence (Fig. 4A). To further elucidate the release of Ox-mtDNA, the mtDNA D-loop, Cox1 and Non-NUMT levels and 8-OH-dG in cytosol were measured. Consistently, after blocking mPTP channel opening by CsA, cytosolic Ox-mtDNA decreased (Fig. 4B, C). The confocal images also showed that CsA increased presence of co-localization between dsDNA and Mito-mCherry in RAW264.7 cells (Fig. 4D). In addition, CsA decreased the expression of cleaved-caspase1 and GSDMD-N in RAW264.7 cells induced by aging microenvironment during H/R (Fig. 4E, F). Furthermore, after treating with CsA, caspase1 activity (Fig. 4H) and the levels of LDH (Fig. 4I), IL-1β and IL-18 (Fig. 4J) in the supernatant were significantly decreased. Morphologically, TEM showed that CsA reduced the membrane discontinuity, cytoplasmic edema, and organelle incompleteness of RAW264.7 cells induced by aging microenvironment during H/R (Fig. 4G). The mitochondrial cristae score was increased after CsA treatment (Supplementary Fig. S4B). To sum up, these results showed that aging induced macrophage pyroptosis by promoting mPTP channel opening thereby increasing the release of Ox-mtDNA.

### Aging enhanced MCU-mediated mitochondrial Ca^2+^ uptake to trigger mPTP channel opening

**Fig. 5:**
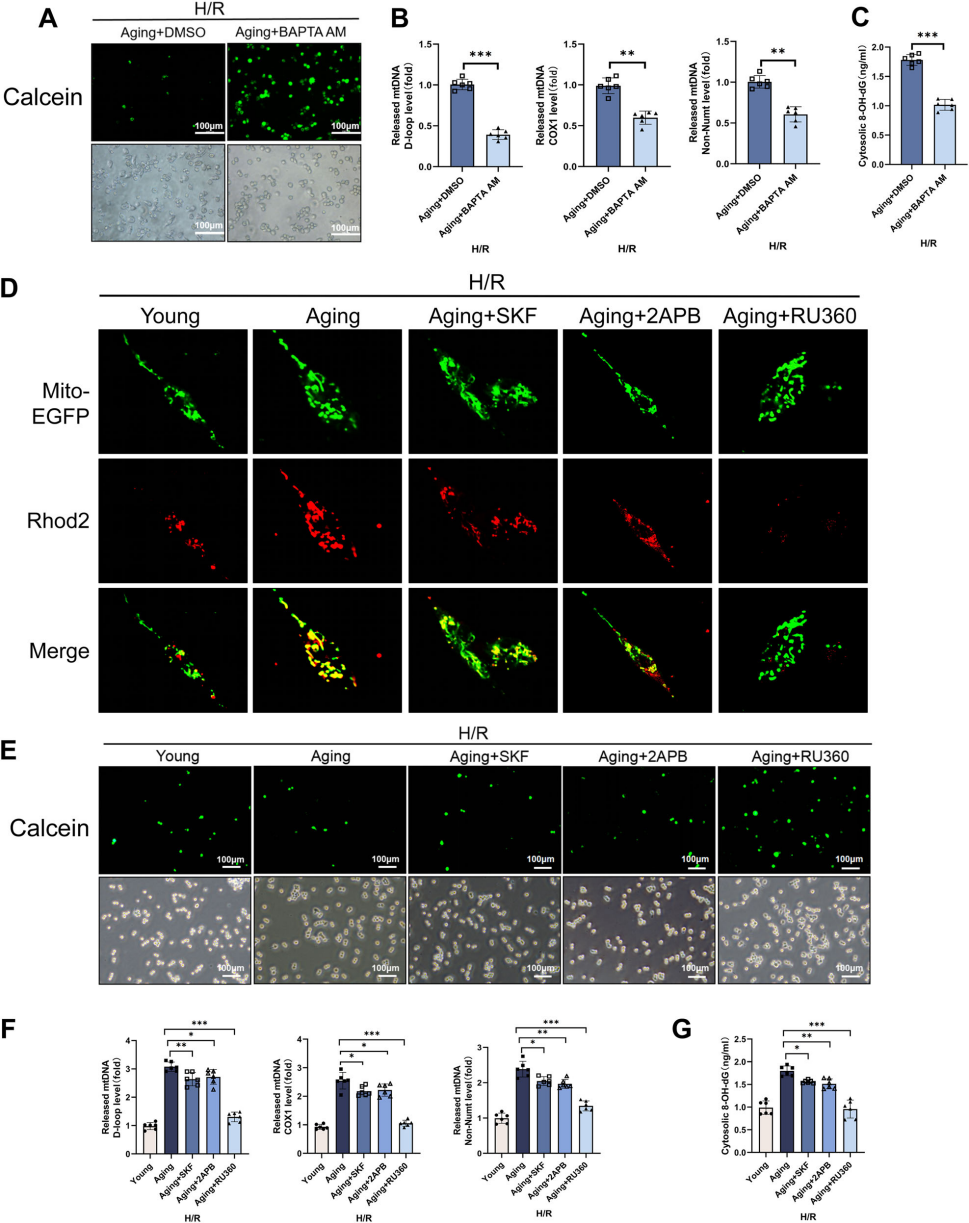
Aging enhanced MCU-mediated mitochondrial Ca^2+^ uptake to trigger mPTP channel opening and Ox-mtDNA release in macrophages during H/R. RAW264.7 cells were co-cultured with the supernatant from normal or aged AML12 cells in the absence or presence of BAPTA-AM (10 μM), SKF (20 μM), 2APB (100 μM), or RU360 (10 μM) for 24 h followed by treatment with H/R. **A** Representative of immunofluorescence staining of Calcein (green) and corresponding cell density (white light). **B** Relative cytosolic mtDNA amounts in each group. The relative ratios of D-loop mtDNA, Cox1 mtDNA, and Non-NUMT mtDNA are tested by qPCR. **C** 8-OH-dG in cytosol were quantified using ELISA. **D** The colocalization of Mito-EGFP (green) and Rhod2 (red) was detected by confocal microscopy. **E** Representative of immunofluorescence staining of Calcein (green) and corresponding cell density (white light). **F** Relative cytosolic mtDNA amounts in each group. The relative ratios of D-loop mtDNA, Cox1 mtDNA, or Non-NUMT mtDNA are tested by qPCR. **G** 8-OH-dG in cytosol were quantified using ELISA. All data are shown as the mean ± SD (*n* = 6). ***p < 0.001, **p < 0.01 and *p < 0.05.

Calcium (Ca^2+^) is a ubiquitous and essential second messenger, and a key factor in triggering mPTP channel opening rather than high level of ROS [18]. The intracellular calcium level is mainly regulated by the release of inositol 1,4,5-trisphosphatereceptor (IP3R) on endoplasmic reticulum (ER) and uptake of calcium uniporter (MCU), as well as the entry of exogenous calcium through the transient receptor potential canonical Channel (TRAC) channel on the cell membrane. Thus, to preliminarily clarify the effect of Ca^2+^ on mPTP channel opening, we used BAPTA-AM (a Ca^2+^ chelator) to inhibit intracellular Ca^2+^ in RAW264.7 cells induced by aging microenvironment during H/R. The fluorescence intensity of Fluo4-AM in the aging group, but decreased significantly after BAPTA-AM treatment, indicating that intracellular Ca^2+^ were chelated (Supplementary Fig. S2). Moreover, BAPTA-AM prevented fluorescence quenching of calcein (Fig. 5A). As shown in Fig. 5B, C, BAPTA-AM reduced cytoplasmic Ox-mtDNA levels. These results implied that Ca^2+^ was a critical factor that aging promoted mPTP channel opening.

Then, to further elucidate the source of mitochondrial Ca^2+^, we treated RAW264.7 cells induced by aging microenvironment during H/R with SKF96365 (SKF, a membrane TRPC channel inhibitor), 2-Aminoethyl Diphenylborate (2APB, an endoplasmic reticulum IP3R inhibitor), or RU360 (a mitochondrial MCU inhibitor), respectively. The confocal images showed that fluorescence of Rhod2 (representing mitochondrial Ca^2+^ binding) in the aging group was strongly elevated. Interestingly, there was no significant change after pretreatment with SKF and 2APB. However, RU360 weakened its fluorescence (Fig. 5D). Consistently, RU360 inhibited the opening of mPTP channel (Fig. 5E) and reduced cytoplasmic Ox-mtDNA levels (Fig. 5F, G), while SKF and 2APB had no significant effect. In summary, these results indicated that in aging microenvironment, aging increased intracellular Ca^2+^ and promoted mitochondrial MCU uptake of Ca^2+^, thereby triggering mPTP channel-mediated Ox-mtDNA release.

### MCU was acetylated in aging-induced macrophages during H/R

**Fig. 6:**
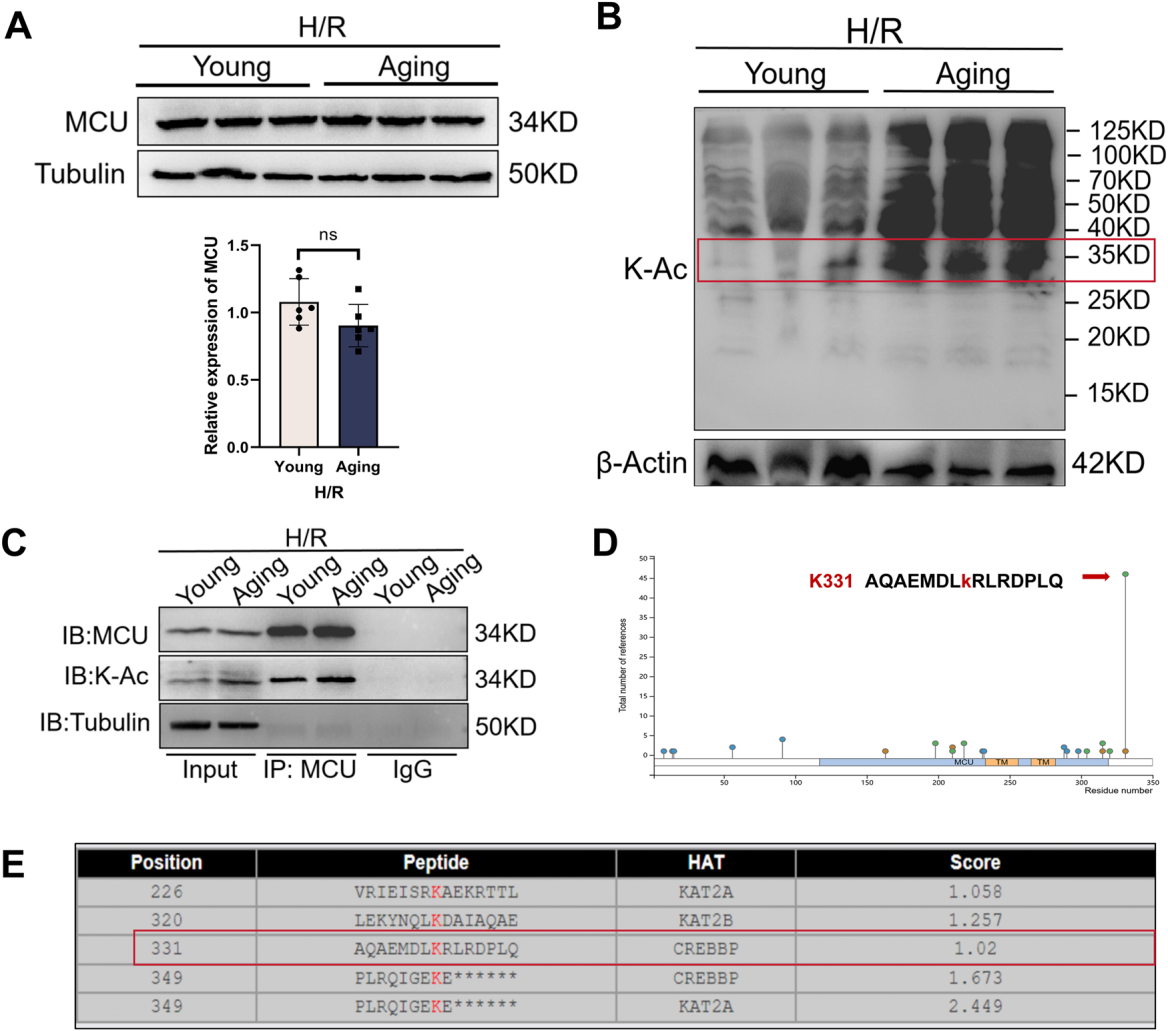
Aging led to acetylation of MCU in macrophages during H/R. RAW264.7 cells were co-cultured with the supernatant from normal or aged AML12 cells for 24 h, followed by treatment with H/R. **A** The level of MCU was measured by WB. **B** Lysine-acetylation (K–Ac) expressions in whole-cell lysates was measured by WB. **C** Co-IP assay was performed to determine MCU acetylation. All data are shown as the mean ± SD (*n* = 6). ***p < 0.001, **p < 0.01 and *p < 0.05. **D** PhosphSitePlus predicted MCU acetylation at K331 site. **E** GPS-PAIL predicted MCU acetylation at K331 site.

We used WB to detect the expression of MCU in RAW264.7 with or without aging microenvironment induction during H/R, but there was no significant difference between the two groups (Fig. 6A). Acetylation is one of the vital protein post translational modifications (PTMs) that impact protein aggregation and protein stability [28]. Given that accumulating evidence has indicated critical roles of protein acetylation in aging, and mitochondria is organelle highly enriched in acetylation besides the nucleus [19, 20, 29], we detected acetylation of whole cells in two groups using WB. Compared with the young group, the acetylation level in the aging group was higher (Fig. 6B). Then, we used Co-IP and found that MCU acetylation in RAW264.7 induced by aging microenvironment during H/R (Fig. 6C). Phosph-SitePlus (Fig. 6D) and GPS-PAIL (Fig. 6E) websites were further used to predict the acetylation site of MCU as K331. These results showed that aging led to acetylation of mitochondrial MCU in macrophages during H/R.

### MCU acetylation at K331 was essential for aging promoting mitochondrial Ca^2+^ uptake, mPTP channel opening and Ox-mtDNA release in macrophages during H/R

**Fig. 7:**
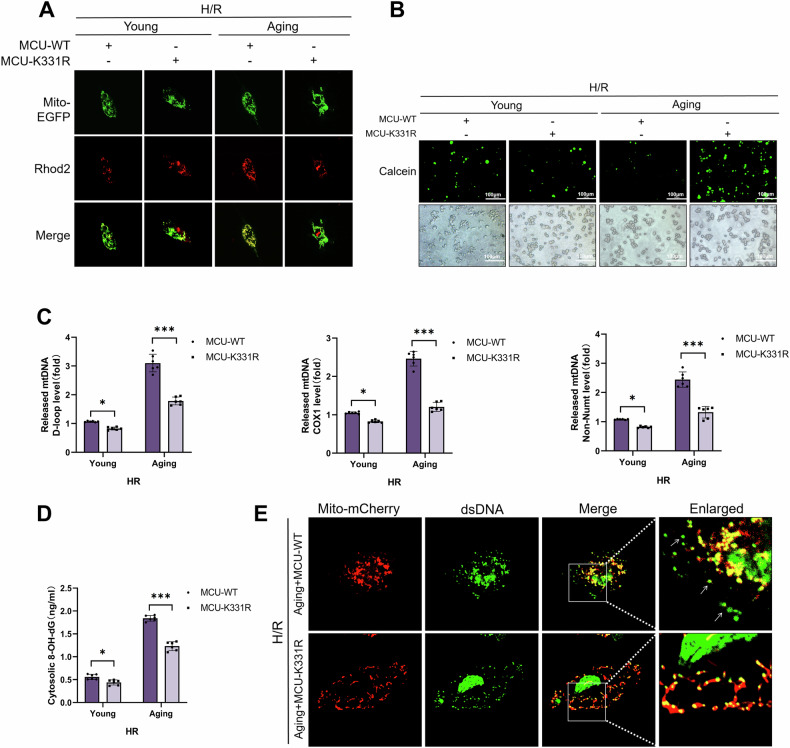
MCU deacetylation restrained mitochondrial Ca^2+^ uptake, mPTP channel opening and Ox-mtDNA release in aging-induced macrophage pyroptosis during H/R. RAW264.7 cells were infected with Lv-MCU-WT or Lv-MCU-K331R and co-cultured with the supernatant from normal or aged AML12 cells for 24 h, followed by treatment with H/R. **A** The colocalization of Mito-EGFP (green) and Rhod2 (red) was detected by confocal microscopy. **B** Representative of immunofluorescence staining of Calcein (green) and corresponding cell density (white light). **C** Relative cytosolic mtDNA amounts in each group. The relative ratios of D-loop mtDNA, Cox1 mtDNA, or Non-NUMT mtDNA are tested by qPCR. **D** 8-OH-dG in cytosol were quantified using ELISA. **E** The colocalization of Mito-mCherry (red) and dsDNA (green) was detected by confocal microscopy. All data are shown as the mean ± SD (*n* = 6). ***p < 0.001, **p < 0.01 and *p < 0.05.

In order to further clarify whether K331 was the site of aging induced MCU acetylation promoting mitochondrial Ca^2+^ overload, mPTP channel opening and Ox-mtDNA, RAW264.7 cells were infected with Lv-MCU-WT or Lv-MCU-K331R before co-culturing with the supernatant from normal or aged AML12 cells and induction by H/R. Co-IP tested and displayed that MCU deacetylation after mutation of K331 (Supplementary Fig. S3). The confocal images showed that during the H/R period, there was no significant difference in the mitochondrial Ca^2+^ level between the young-MCU-MT group and young-MCU-K331R group. However, the level of mitochondrial Ca^2+^ in aging-MCU-K331R group was lower than that in aging-MCU-MT group (Fig. 7A). Similarly, compared with the aging-MCU-WT group, the aging-MCU-K331R group showed enhanced fluorescence of calcein (Fig. 7B) and decreased cytoplasmic Ox mtDNA (Fig. 7C, D). However, compared with the young-MCU-WT group, the young-MCU-K331R group showed slight changes in the enhancement of calcein (Fig. 7B) and the reduction of cytoplasmic Ox mtDNA (Fig. 7C, D). In addition, after aging-induced RAW264.7 cells were treatment with MCU K331R mutant, co-localization of dsDNA and mitochondria was increased (Fig. 7E). These results indicated that aging induced MCU acetylation at K331, resulting in mitochondrial Ca^2+^ overload, mPTP channel opening and Ox-mtDNA of macrophage during H/R.

### MCU acetylation at K331 was vital for aging facilitating macrophage pyroptosis during H/R

**Fig. 8:**
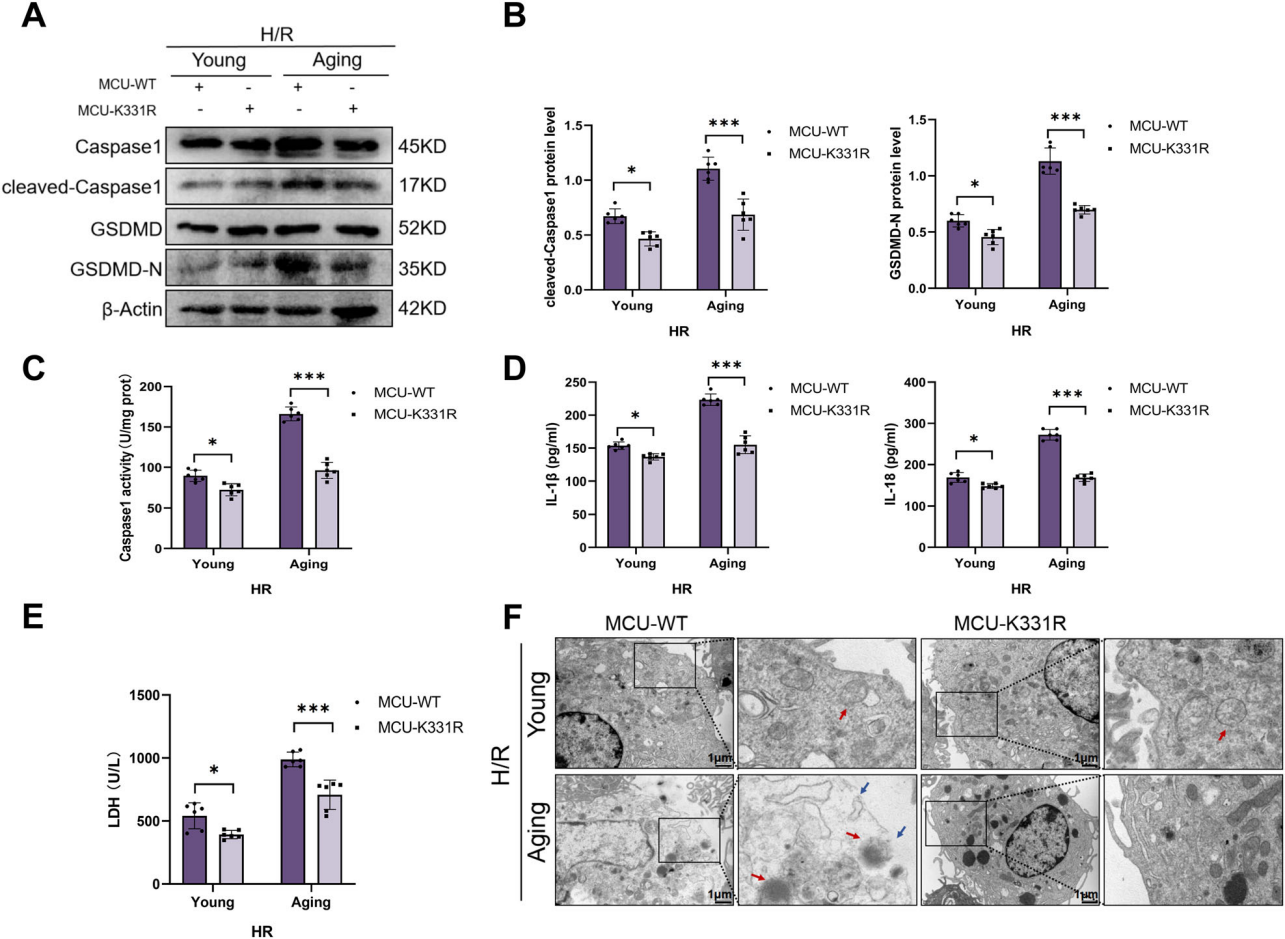
MCU deacetylation alleviated aging-induced macrophage pyroptosis during H/R. RAW264.7 cells were infected with LV-MCU-WT or LV-MCU-K331R and co-cultured with the supernatant from normal or aged AML12 cells for 24 h, followed by treatment with H/R. **A**, **B** The levels of Caspase1, Cleaved-Caspase1, GSDMD and GSDMD-N were detected by WB. **C** Caspase1 activity was tested by the caspase1 assay kit. **D** The levels of IL-1β and IL-18 in the cell culture supernatant were measured by ELISA. **E** Supernatant LDH levels were measured. **F** TEM was used to observe the ultrastructural changes in macrophages (magnification, 12,000×). The red arrow indicates the incomplete structure of an organelle, and the blue arrow indicates discontinuity in the cell membrane. All data are shown as the mean ± SD (*n* = 6). ***p < 0.001, **p < 0.01 and *p < 0.05.

We continued to investigate the effect of MCU K331R mutant on macrophage pyroptosis with ot without aging microenvironment induction during H/R. WB showed that in the young group, the mutation of MCU K331R only slightly reduced the expression of cleaned-caspase1 and GSDMD-N, but in the aging group, the mutation of MCU K331R significantly reduced the expression of cleaned-caspase1 and GSDMD-N (Fig. 8A, B). Furthermore, compared with the young group, the aging group showed that after treatment with MCU K331 mutant, a more significant decrease in caspase1 activity (Fig. 8C), supernatant IL1β, IL-18 (Fig. 8D), and LDH (Fig. 8E) in RAW264.7 cells. TEM showed that compared with the young group, after treatment with MCU K331 mutant in aging group, RAW264.7 cells had a more significant reduction in edema, organelle damage, and incompletion of cell membrane (Fig. 8F). Moreover, in the aging group, the mitochondrial cristae score was significantly increased after treatment with MCU K331 mutant (Supplementary Fig. S4C). In brief, MCU acetylation at K331 was an important factor for aging inducing macrophage pyroptosis during H/R.

## Discussion

Older donor age is an important factor for decrease of survival after re-transplantation [4, 30], and ischemia reperfusion injury (IRI) is a major cause of graft dysfunction during liver transplantation for aged donor [31, 32]. Interestingly, a study shows that the impact of donor age on post-transplant mortality becomes evident in donors as young as 40 years of age and escalates progressively thereafter [33]. The 1-year post-transplant survival rate for donors under 40 years old is 85–86%, but for donors over 70 years old, the 1-year post-transplant survival rate is only 61–76% [33]. However, the mechanism of the decrease survival rate caused by aging has not been fully elucidated. Therefore, we used young and aged mice and constructed ischemia for 90 min and reperfusion for 6 or 24 h, respectively. We found that after 24 h of reperfusion, liver damage in young mice gradually recovered, but in aged mice, the damage was still severe. This result preliminarily revealed that aging delayed the recovery of liver function after IRI.

Aging is characterized by systemic chronic inflammation, which is accompanied by cellular senescence, immuno-senescence, organ dysfunction, and age-related diseases [34]. Pyroptosis, an inflammation dependent programmed cell death, was first discovered in 1992 [35] and named “pyroptosis” in 2001 [36]. It is mainly divided into caspase 1-mediated classic pathways and caspases 4, 5, and 11-mediated nonclassical pathways [37]. During pyroptosis, cells experience cytoplasmic swelling, organelle deformation, and cleavage of Gasdermin D (GSDMD) by activated caspases, thereby producing the “GSDMD-N” fragment. This fragment forms pores in the plasma membrane, leading to the release of significant amounts of LDH, IL-1β, and IL-18, which further exacerbate the inflammatory response [37]. In recent years, more and more studies have shown that macrophage pyroptosis is one of the important factors leading to the occurrence and development of aging diseases [12, 13]. However, it is unclear whether aging promotes macrophage pyroptosis during liver IRI. In this study, we found that after 24 h of reperfusion, the degree of macrophage pyroptosis worsened in aged mice, suggesting that macrophage pyroptosis was an important factor for aging delaying liver function after IRI.

The accumulation and release of oxidized mtDNA are thought to contribute to aging-induced cell death, senescence and tissue dysfunction [26, 38]. Ox-mtDNA fragments release from stressed mitochondria to activate the DNA-sensing cyclic GMP-AMP (cGAMP) synthase (cGAS)-stimulator interferon genes (STING) pathway and NLRP3 [39]. The channels through which damaged mtDNA is released into the cytoplasm mainly include the BCL2-Associated X Protein (BAX)/Bcl2 Antagonist/Killer 1 (BAK) pore and the mPTP channel [40, 41]. However, some studies reported that mtDNA leakage through Bax/Bak pores to promote apoptosis pathway, but activation of the mPTP channel is associated with the release of mitochondrial constituents, including mtDNA (up to 700 bp in length) to induce inflammatory responses [40, 41]. Accordingly, we used the mPTP channel inhibitor to close the channel opening and tested macrophage pyroptosis. The results proved that aging increased the mPTP-mediated Ox-mtDNA release to induce macrophage pyroptosis during H/R. However, there are a few studies indicating that mitochondrial derived vesicles (MDVs) [42] and mitochondrial fission [43] also mediated mtDNA release. Whether aging promotes mtDNA release through other pathways to induce macrophage pyroptosis will continue to be studied in the future.

Regarding the activation mechanism of mPTP channel, although research indicated that high levels of Ca^2+^ in the absence of ROS induces mPTP channel opening regulated by both CypD (cyclophilin D) and ANTs (adenine nucleotide translocators) [18], the level of intracellular Ca^2+^ is mainly regulated by extracellular Ca^2+^ influx through TRPC channel [44, 45], Ca^2+^ release through the inositol-1,4,5-trisphosphate receptor (IP3R) channel from endoplasmic reticulum (ER) [46, 47], and Ca^2+^ uptake by mitochondrial calcium uniporter (MCU) [48]. The activation mechanism of the mPTP channel by Ca^2+^ is currently not fully elucidated. Accordingly, we separately used the inhibitor of TRPC, IP3R, and MCU and explored the activity of the mPTP channel and Ox-mtDNA release. We demonstrated that aging promoted mitochondrial MCU uptake of Ca^2+^, thereby triggering mPTP channel-mediated Ox-mtDNA release to induce macrophage pyroptosis. In addition to increased inflammation, other studies indicate that a reduction in mitochondrial Ca^2+^ uptake also affects the ability of macrophages to phagocytose and kill pathogens, an immunological deficit known to be related to age. But this pathological mechanism has not yet been fully elucidated [49–51].

As a key post-translational modification in mammalian cells, lysine acetylation regulates numerous molecular processes. Significantly, elevated mitochondrial protein acetylation levels are a recognized feature of the aged liver [20, 21, 52, 53]. In this study, we demonstrated that aging induced acetylation of MCU at K331 in macrophages, and MCU acetylation at K331 acts as a critical mediator, promoting increased mitochondrial Ca^2+^ uptake. This enhanced uptake subsequently stimulates the release of oxidized mtDNA (Ox-mtDNA) through the mPTP channel activity, ultimately leading to macrophage pyroptosis. Mitochondria serve as the acetylation-enriched place in except for nucleus, because acetyl coenzyme A (CoA) accumulates highly in mitochondria and participates in the tricarboxylic acid (TCA) cycle [54]. Besides, Proteins in mitochondria seem to be easily acetylated through non enzymatic mechanisms [54]. The sirtuin (SIRT) family is a group of deacetylases consisting of SIRT1-7. Three members of sirtuin family, SIRT3, SIRT4, and SIRT5 are specific in mitochondria, SIRT2 is located in the cytoplasm, but SIRT1 is mainly located in nucleus [55]. Among them, studies have reported that SIRT1inhibition increases the acetylation of MCU, resulting in mitochondrial Ca^2+^overload [54]. On the other hand, SIRT3 inhibits mitochondrial calcium uniporter (MCU)-mediated mitochondrial calcium overload by reducing the H3K27ac level on the MCU promoter in an AMPK-dependent manner [56]. Accordingly, SIRT1 and SIRT3 may play an important regulatory role in MCU acetylation in aged liver.

## Conclusion

This study found that aging induced macrophage pyroptosis to delay the recovery of liver function after IRI. Mechanistically, it highlighted that aging triggered the opening of the mPTP channel to increase Ox-mtDNA release, thereby inducing macrophage pyroptosis. In addition, it further elucidated the activity of the mPTP channel was mainly dependent on calcium uptake by acetylated MCU at K331. In summary, these results clarified that cytoplasmic Ox-mtDNA-induced macrophage pyroptosis was a key factor for aging deferring liver function restoration. Calcium uptake via acetylated MCU triggered mPTP channels opening, which is an important mechanism for Ox-mtDNA release from mitochondria into the cytoplasm.

## Materials and methods

### Materials

Antibodies against Acetyl lysine (ab21623), Caspase1(ab179515), GSDMD (ab209845), p16 (ab51243) and p21 (ab188224) were purchased from Abcam (Cambridge, UK). Antibodies against Caspase1 (HA722222), Cox4 (ET1701-63) and β-Actin (M1210-2) were purchased from Huabio (Shandong, China). MCU(#14997S) was purchased from CST (Boston, USA). Tubulin(AFRM9332) was purchased from AiFang Biological (Hunan, China). LV-MCU-WT and LV-MCU-K331R were purchased from Hanbio(Shanghai, China). 2-Aminoethyl Diphenylborate (2APB), BAPTA-AM, Cyclosporin A (CsA) and SKF96365 were purchased from Selleck (Texas, USA). RU360 was purchased from GLPBIO (Montclair, USA). Plvw-CMV-Mito-mCherry-EF1-puro and Plvw-CMV-Mito-EGFP-EF1-puro were purchased from Leapwal biotech (Hunan, China). dsDNA (S11494) was purchased from Invitrogen. Etoposide (HY-13629) and Rhod2-AM (HY-D0989) were purchased from MCE (New Jersey, USA). Lacate dehydrogenase (LDH) kit (BC0685), Alanine aminotransferase (AST) kit (BC1565), aspartate transaminase (ALT) kit (BC1555) and the caspase1 activity assay kit (BC3810) were purchased from Solarbio (Beijing, China). Enzyme-linked immunosorbent assay (ELISA) kits for IL-1β (QZ-10489) and IL-18 (QZ-10246) were obtained from Jiubang Biotechnology (Quanzhou, China). Primers for D-loop, Cox1, Non-NUMT were provided by Sangon Biotech (Shanghai, China). Fluo4-AM (S1060) and mPTP channel kit (C2009S) were purchased from Beyotime Biotechnology (Shanghai, China). 8-hydroxy 2 deoxyguanosine (8-OH-dG) ELISA kit (ab201734) was purchased from abcam. RAW264.7 cells and AML12 cells were purchased from Pricella (Wuhan, China), and these cells used were identified by STR and tested for cytoplasm contamination.

### Animals

Young (8–10 weeks) and aged (18 months) male wild-type (WT) C57BL/6 mice were purchased by the Experimental Animal Center of Chongqing Medical University (Chongqing, China). The mice were randomly allocated into the experimental and control groups to ensure unbiased distribution. Humane care guided by the guidelines of the National Institutes of Health was provided to all animals. The protocols used in this research were evaluated and approved by the Animal Use and Ethics Committee of the 2nd Affiliated Hospital of Chongqing Medical University (IACUC-CQMU-2025-0252).

### Liver IRI model

A model of partial warm hepatic IRI was established as described previously [57]. In brief, for young and aged mice, the sham group had only free hepatic portal blood vessels after laparotomy without blockade of blood flow. The IRI group had the hepatic portal vein clamped, which blocked the blood supply to the left lobe and midhepatic lobe, for 90 min, and the blood vessels were then opened for 6 h or 24 h. If a mouse died before a sample was collected, the sample was discarded. All operations were performed by the same operator, and the mice were fasted for 12 h before surgery.

### Liver damage assessment

Serum ALT and AST levels were measured by a micromethod according to the manufacturer’s instructions. Some liver specimens were fixed in 4% paraformal-dehyde and embedded in paraffin. Liver sections were stained with hematoxylin and eosin (H&E). The severity of liver IRI was graded using the Suzuki score. Tissues without necrosis or congestion/ centrilobular ballooning were given a score of 0, whereas those presenting with severe congestion and/or >60% lobular necrosis were given a score of 4.

### Cell culture and treatment

#### Isolation and cultivation of liver macrophages

According to the three-step approach proposed by Li [58] that includes digestion with collagenase IV (Sigma-Aldrich), gradient centrifugation and selective adherence, liver macrophages were isolated from normal liver samples. The liver macrophages were then cultured in DMEM (Gibco) supplemented with 10% FBS (Gibco), 100 U/mL penicillin G (Beyotime) and 100 U/mL streptomycin (Beyotime) at 37 °C in the presence of 5% CO_2_.

#### Passage and cultivation of RAW264.7 cells

The culture medium of RAW264.7 cells was discarded. Cells were added DMEM and gently blowed off until cells fell off. After centrifugation (1000 rpm, 5 minutes), the supernatant was discarded. RAW264.7 cells were cultured in DMEM (Gibco) supplemented with 10% FBS (Gibco), 100 U/mL penicillin G (Beyotime) and 100 U/mL streptomycin (Beyotime) at 37 °C in the presence of 5% CO_2_.

#### Passage and cultivation of AML12 cells

The culture medium of AML12 cells was discarded and added 0.25% trypsin digestion solution for 1–2 min until most of the cells become round and shed. Stop digestion by adding complete culture medium. After centrifugation (1000 rpm, 5 min), the supernatant was discarded. AML12 cells were cultured in DMEM/F12 (Gibco) supplemented with 10% FBS (Gibco), 0.5% ITS-G (100×, Beyotime), 40 ng/ml Dexamethasone (Beyotime), 100 U/mL penicillin G (Beyotime) and 100 U/mL streptomycin (Beyotime) at 37 °C in the presence of 5% CO_2_.

#### Co-culture of RAW264.7 cells and AML12 cells

AML12 cells were treated with Etoposide (75 μM) or equal volume of DMSO for 3 days to simulate as aged or young hepatocytes in vitro, respectively. Then, the supernatant of the two groups of AML12 cells were separately added to RAW264.7 cells culture dishes and co-cultured for 24 h.

#### H/R treatment

To perform H/R in vitro, RAW264.7 cells were cultured at 37 °C in an incubator chamber with an atmosphere of 1% O_2_, 5% CO_2_ and 94% N_2_ for 6 h. Then RAW264.7 cells were returned to a normoxic incubator for 24 h.

#### Drug administration and experimental design in vitro

To block the mPTP channel opening, RAW264.7 cells were treated with CsA (1 μM, 24 h) before H/R.

To suppress intracellular Ca^2+^, RAW264.7 cells were treated with CsA (10 μM, 24 h) before H/R.

To inhibit cell membrane, endoplasmic reticulum, or mitochondrial Ca^2+^ channels, RAW264.7 cells were treated with SKF96365 (20 μM), 2APB (100 μM), RU360 (10 μM) for 24 h, respectively, followed by H/R treatment.

#### Lentivirus (Lv) infection

Using the 1/2 small volume infection method, RAW264.7 cells were added to 1/2 fresh complete culture medium, Lv-MCU-WT or Lv-MCU-K331R were transfected into the cell according to cell density (MOI = 30), and an appropriate amount of infectious fluid was added. After 4 h, the remaining 1/2 complete culture medium was supplemented. Fresh complete culture medium was replaced after 24 h. Screening of cells successfully transfected with Lv using puromycin (5 μg/ml, Beyotime).

### Immunohistochemical staining (IHC)

The paraffin-embedded sections were placed in an oven at 60 °C and then immersed in xylene and a series of ethanol solutions to remove the paraffin. The slides were then placed in Tris-EDTA antigen retrieval solution (Beyotime) and heated in a microwave for 8 min on medium-high power followed by 20 min on medium-low power, and left to cool to room temperature. The sections were then incubated with 3% hydrogen peroxide for 10 min at room temperature to block endogenous peroxidase activity. After washing with PBS, the sections were incubated with caspase1 antibody (1:500, Huabio) at 4 °C for over 12 h. Following incubation, excess antibody was washed off with PBS, and the sections were treated with reagents from an IHC kit (including the enhancer and IgG polymer, AiFang Biological) at room temperature. The sections were then stained with 3,3′-diaminobenzidine (DAB) and counterstained with haematoxylin. Subsequently, the slides were dehydrated by sequential immersion in increasing ethanol concentrations and xylene. Finally, the slides were mounted with neutral gum and examined under a light microscope.

### Transmission electron microscopy (TEM)

Liver tissues harvested from mice were cut into 1-mm3 pieces and fixed in 3% glutaraldehyde. RAW264.7 cells were centrifuged (12,000 rpm, 10 min), and then fixed with 3% glutaraldehyde. These specimens were delivered to the Electron Microscopy Center of Chengdu Lilai Biotechnology Co., Ltd and observed under TEM.

To evaluate a cristae score of mitochondrion, assess both the density and structural integrity of the cristae. Assign a score on a scale from 0 to 4 according to the following criteria (0—no sharply defined crista, 1—greater than 50% of the mitochondrial area without cristae, 2—greater than 25% of mitochondrial area without cristae, 3—manycristae (over 75% of area) but irregular, 4—many regular cristae) [59].

### Western blot (WB)

RIPA lysis buffer (Beyotime) containing 1× mixture of protease phosphatase inhibitors (Beyotime) was used to prepare protein samples from cells or tissues. The lysates were centrifuged for 15 min at 12,000 rpm at 4 °C and then the supernatant was collected and quantified by the BCA Protein Assay Kit (Beyotime). After adding 5x SDS-PAGE loading buffer and boiling at 100 °C for 10 min, equal amounts of protein were run on an SDS-PAGE gel. After being electrophoresed and transferred to a PVDF membrane, the membrane was blocked for 1 h in 5% BSA and incubated with primary antibody overnight at 4 °C. The following primary antibodies were used: p16 (1:1000), p21 (1:1000), MCU (1:1000), caspase1, cleaved-caspase1 (1:1000), GSDMD, GSDMD-N (1:1000), Cox4 (1:1000), β-actin (1:10000) and tubulin (1:8000). For detecting acetylation expression, protein was transferred to a NC membrane, the membrane was blocked for 1 h in 5% non-fat powdered milk (Beyotime) and incubated with Acetyl lysine (1:1000) primary antibody overnight at 4 °C. Then, Both PVDF and NC membranes were incubated with secondary antibodies (1:8000, BOSTER) for 1 h at room temperature. The bands were visualized with the image system (Bio-Rad, Universal Hood III). Relative density of the protein immunoblot images was analyzed by Image Lab software (Image Lab 3.0, USA).

### Co-Immunoprecipitation (Co-IP)

RAW264.7 cells were lysised and centrifuged. Measure the protein concentration using the BCA Protein Assay Kit. Divide each sample evenly into the Input group, the IP group, and the IgG group. Add 5x SDS-PAGE loading buffer to the Input group, and then cook the protein at 100 °C for 10 min. Add MCU antibody (1:50) to the IP group and equal concentration of rabbit IgG antibody (CST) to the IgG group. Then slowly shake at 4 °C overnight. On the second day, Protein A/G Magnetic Beads (MCE) were added to the IP group and the IgG group according to the manufacturer’s instructions. Finally, add 1x SDS-SPAGE loading buffer (Biyotime) and then cook the protein at 100 °C for 10 min. Western blotting was performed as previously described.

### Extraction of mitochondria

First, prepare the HB buffer by combining HEPES buffer, sucrose solution, and sterile ddH2O to achieve a final concentration of 250 mM sucrose and 10 mM HEPES, with a pH of 7.4. 1× mixture of protease phosphatase inhibitors were added, and the solution was placed on ice. Liver macrophages or RAW264.7 cells were collected and resuspended in 1 mL of pre-cooled HB buffer. After 10 minutes, a 1 mL insulin syringe equipped with a 22 G insulin needle was used to repeatedly aspirate the cell suspension 30 times on ice. The cell homogenate was then centrifuged at 1100 g for 10 min at 4 °C. The supernatant represented the whole cell extracts, while the pellet was discarded. Next, the supernatant was centrifuged for 15 min at 11,000 × *g* at 4 °C. The supernatant represented the cytosolic fractions. The pellet was resuspended in 1 mL of HB buffer, and then centrifuged for 15 min at 11,000 × *g* at 4 °C. The supernatant is discarded, and the pellet is retained. The crude mitochondrial fraction was then gently resuspended in 100–150 µL of HB buffer. Protein concentration was determined using the BCA kit. The successful isolation of mitochondria was confirmed by Western blot analysis of β-actin and Cox4 expression.

### Measurement of mtDNA

After DNA purification with BeyoMag™ Magnetic bead PCR/DNA purification kit (D0041, Beyotime) according to manufacturer’s instructions, from both whole cell extracts and cytosolic fractions, mtDNA was quantified by RT-qPCR using the SYBR Green qPCR Master Mix (No ROX, HY-K0523, MCE) and primers specific for the mitochondrial D-loop region, cytochrome coxidase (Cox1) and a specific region of mtDNA that was not inserted into nuclear DNA (Non-NUMT). The Ct values obtained for mtDNA abundance in whole cell extracts served as normalization controls for the mtDNA values obtained from the cytosolic fractions.

The primers used for RT-PCR analysis were as follows:

D-loop(mouse) forward: GTGTTATCTGACATACACCATACAG,

D-loop (mouse) reverse: TGGGAACTACTAGAATTGATCAGGA.

Cox1 (mouse) forward: GCCCCAGATATAGCATTCCC,

Cox1 (mouse) reverse: GTTCATCCTGTTCCTGCTCC.

Non-NUMT (mouse) forward: CTAGAAACCCCGAAACCAAA,

Non-NUMT (mouse) reverse: CCAGCTATCACCAAGCTCGT.

### Measurement of oxidized mtDNA (Ox-mtDNA)

For the measurement of Ox-mtDNA, purified mtDNA was extracted from the cytosolic or mitochondrial fractions as indicated above. The 8-OH-dG content was then quantified using the 8-OH-dG ELISA kit.

### Co-localization of mitochondria and dsDNA

As indicated above, using the 1/2 small volume infection method, RAW264.7 cells were infected with Plvw-CMV-Mito-mCherry-EF1-puro (Red). Detection of transfection efficiency through fluorescence microscopy. According to the manufacturer’s instructions, SYBR™ gold nucleic acid gel fuel (dsDNA staining, green) was diluted at 1:10,000, and incubated for 30 min at 37 °C, and then PBS washed three times before the confocal imaging.

### Measurement of mitochondria Ca^2+^

As indicated above, using the 1/2 small volume infection method, RAW264.7 cells were infected with Plvw-CMV-Mito-EGFP-EF1-puro (Green). Detection of transfection efficiency through fluorescence microscopy. According to the manufacturer’s instructions, cells were incubated with Rhod-2 AM (2 μM) for 30 min at 37 °C in the dark, and then PBS washed three times before the confocal imaging.

### Measurement of intracellular Ca^2+^

According to the manufacturer’s instructions, RAW264.7 cells were treated with Fluo-4 AM (1 μM) at 37 °C for 30 min in the dark. The fluorescence intensity of cells was observed intracellular calcium signal with the inverted fluorescence microscope.

### Measurement of mPTP channel opening

According to the manufacturer’s instructions, RAW264.7 cells were treated with 1 × calcein and 1 × CoCl_2_ for 30 min at 37 °C in the dark, and then PBS washed three times before the inverted fluorescence microscope.

### Caspase1 activity

Caspase1 activity in liver macrophages and RAW264.7 were measured with the caspase1 assay kit according to the manufacturer’s instructions. Briefly, cells were lysed and centrifuged at 15,000 × *g* for 15 min at 4 °C to collect the supernatant. The reaction mixture, containing 40 μL Reagent I, 50 μL sample, and 10 μL Reagent III, was incubated at 37 °C for 100 min. Measure the absorbance value at 405 nm. A standard curve was generated using serial dilutions of pNA (0–200 μmol/L) for calibration.

### Enzyme-linked immunosorbent assay (ELISA) for IL-1β and IL-18

ELISA kits were used to detect IL-1β and IL-18 in mouse serum or RAW264.7 cells supernatant according to the manufacturer’s protocols. Following the manufacturer’s instructions, 50 μL of the test sample was added to the sample wells, followed by 100 μL of HRP-conjugated detection antibody. The plate was sealed and incubated at 37 °C for 60 min. After discarding the liquid, the wells were washed five times with washing buffer. Then, 50 μL of substrates A and B was added, and the plate was incubated at 37 °C for 15 min. After adding 50 μL of stop solution, the OD at 450 nm was measured within 15 min. The sample concentrations were calculated using the standard curve.

### LDH assays

LDH in mouse serum or RAW264.7 cells supernatant was quantitated by the LDH activity assay kit according to the manufacturer’s instructions. Briefly, the mouse serum or supernatant (10 μL) were incubated with 50 μL Reagent I and 10 μL Reagent II at 37 °C for 15 min. Next, 50 μL of Reagent III was added, followed by 15 min incubation at 37 °C. Finally, 150 μL of Reagent IV was added, and the mixture was kept at room temperature for 3 min. Measure the absorbance value at 405 nm. A standard curve was generated using serial dilutions of pyruvate (0–2 μmol/mL).

### Prediction of MCU acetylation sites

MCU acetylation sites were elucidated using the PhosphoSitePlus database (http://www.phosphosite.org/) and GPS-PAIL database (http://www.pail.biocuc-koo.org/).

### Statistical analysis

All results were analyzed using SPSS 18.0 software (SPSS Inc., Chicago, USA). Normally distributed data are shown as the mean ± SD. Differences between groups were evaluated using a *t* test. The Shapiro–Wilk test was used to test for normality. Data exhibiting a *p* value > 0.05 were regarded as conforming to a normal distribution. Nonnormally distributed data are shown as the median, and differences were evaluated using the rank-sum test. Differences with *p* values < 0.05 were regarded as statistically significant.

## Supplementary information

Supplementary legends and figures

Dataset 1

## Data Availability

All data included in this study are available upon request by contacting the corresponding author.
