# Supplementary material for: Aging aggravated liver ischemia and reperfusion injury by promoting oxidized mtDNA mediated-macrophage pyroptosis through acetylated MCU-dependent calcium uptake
Source: Cell Death Discov. 2025 Oct 7;11:449. doi: 10.1038/s41420-025-02746-9 (PMC12504438; doi:10.1038/s41420-025-02746-9)
Supplement: Supplementary file 1 — Supplementary legends and figures [file 41420_2025_2746_MOESM1_ESM.pdf]

Supplementary figure legends

Fig. S1

A. Isolation and extraction of whole cell lysate, mitochondria, and cytoplasm from liver macrophages. **B-C.** AML12 cells were treated with Etoposide (75 μM) or equal volume of DMSO for 3 days to simulate as aged or young hepatocytes in vitro, respectively. The levels of P16 and P21 in AML12 cells with or without etoposide were measured by WB. **D.** The protocol of in vitro experiments for co-culture of AML12 cells and and RAW264.7 cells. RAW264.7 cells were co-cultured with the supernatant from normal or aged AML12 cells. **E.** Isolation and extraction of whole cell lysate, mitochondria, and cytoplasm from RAW264.7 cells. All data are shown as the mean ± SD (n=6). ***$p < 0.001$, **$p < 0.01$ and *$p < 0.05$.

Fig. S2

RAW264.7 cells were co-cultured with the supernatant from normal or aged AML12 cells in the absence or presence of BAPTA-AM (10 μM) for 24 h followed by treatment with H/R: Fluo4-am detects intracellular calcium levels. (n=6)

Fig. S3

RAW264.7 cells were infected with Lv-MCU-WT or Lv-MCU-K331R and co-cultured with the supernatant from normal or aged AML12 cells for 24 h, followed by treatment with H/R: Co-IP assay was performed to determine MCU acetylation. (n=6)

Fig. S4

Quantify mitochondrial cristae based on transmission electron microscope (TEM) images. **A.** Liver in young and aged mice were subjected to ischemia for 90 min followed by reperfusion for 24 h. **B.** RAW264.7 cells were co-cultured with the supernatant from normal or aged AML12 cells in the absence or presence of CsA (1 μM) for 24 h followed by treatment with H/R. **C.** RAW264.7 cells were infected with LV-MCU-WT or LV-MCU-K331R and co-cultured with the supernatant from normal or aged AML12 cells for 24 h, followed by treatment with H/R. All data are shown as the mean ± SD (n=6). ***$p < 0.001$, **$p < 0.01$ and *$p < 0.05$.

Fig. S1

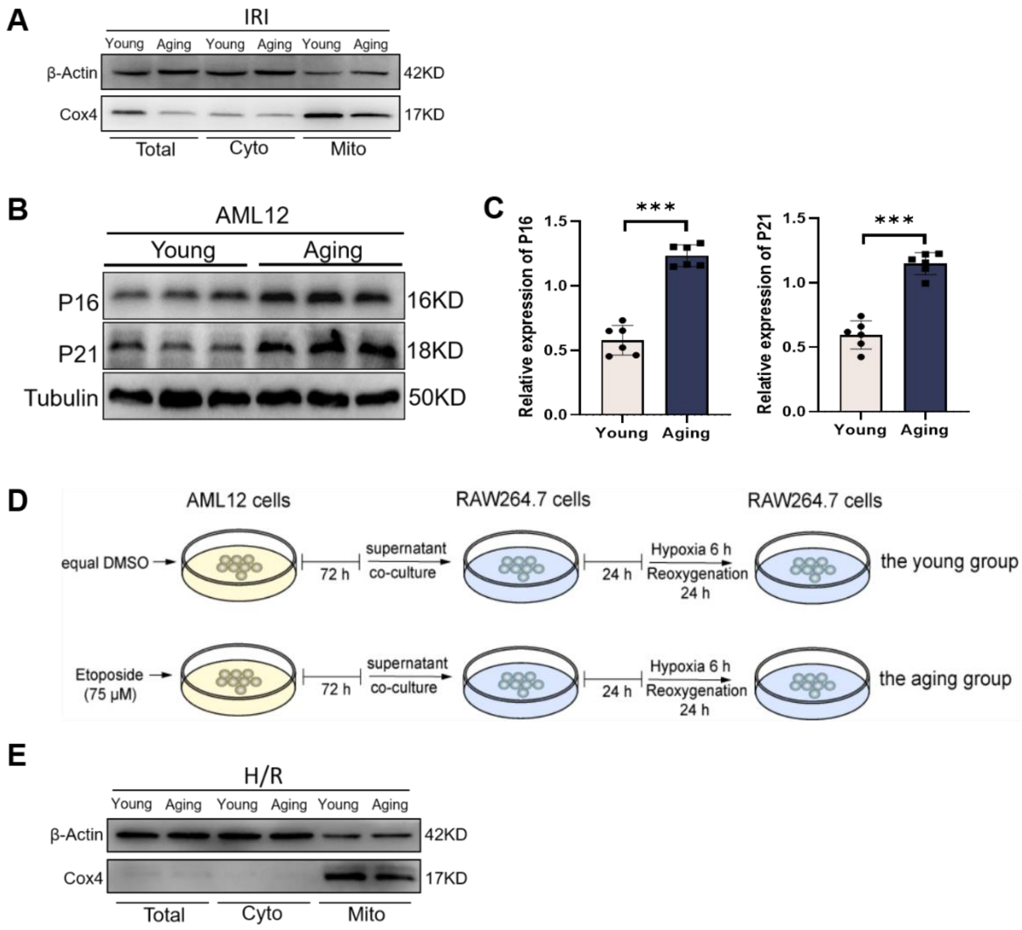

A
IRI
Young Aging Young Aging Young Aging
β-Actin 42KD
Cox4 17KD
Total Cyto Mito
B
AML12
Young Aging
P16 16KD
P21 18KD
Tubulin 50KD
C
***
Relative expression of P16
Relative expression of P21
Young Aging
D
AML12 cells
RAW264.7 cells
RAW264.7 cells
equal DMSO
Etoposide (75 μM)
72 h
supernatant co-culture
24 h
Hypoxia 6 h Reoxygenation 24 h
the young group
the aging group
E
H/R
Young Aging Young Aging Young Aging
β-Actin 42KD
Cox4 17KD
Total Cyto Mito

Fig. S2

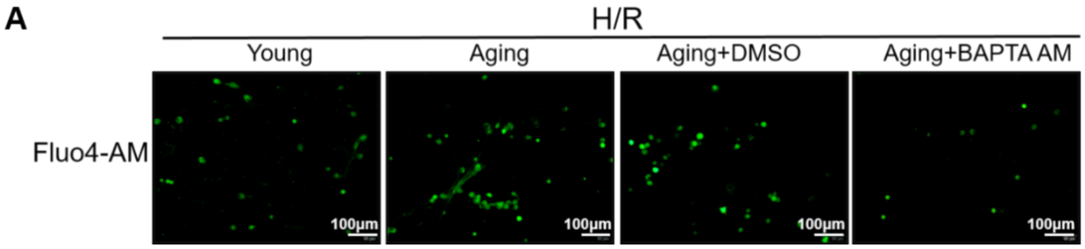
A
H/R
Young
Aging
Aging+DMSO
Aging+BAPTA AM
Fluo4-AM
100μm
100μm
100μm
100μm

Fig. S3

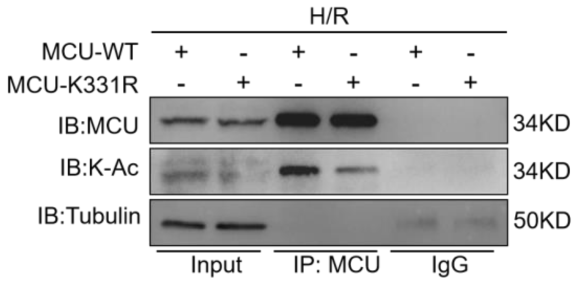
H/R
MCU-WT + - + - + -
MCU-K331R - + - + - +
IB:MCU
34KD
IB:K-Ac
34KD
IB:Tubulin
50KD
Input
IP: MCU
IgG

Fig.S4

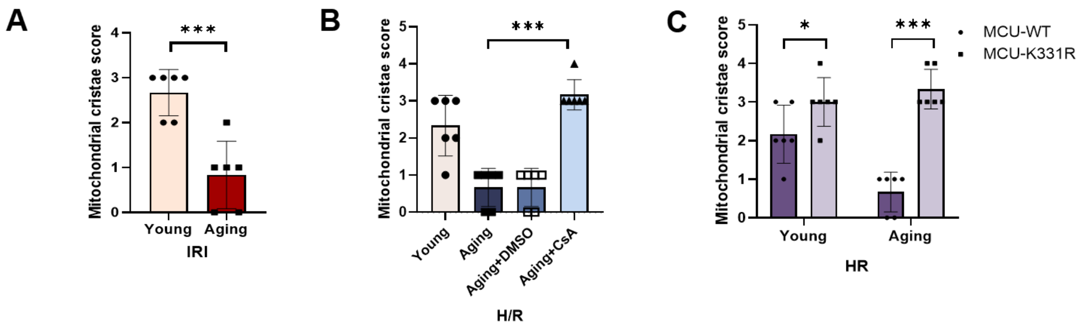

A
Mitochondrial cristae score
***
Young
Aging
IRI
B
Mitochondrial cristae score
***
Young
Aging
Aging+DMSO
Aging+CsA
H/R
C
Mitochondrial cristae score
*
***
MCU-WT
MCU-K331R
Young
Aging
HR
