# Supplementary material for: Aging aggravated liver ischemia and reperfusion injury by promoting oxidized mtDNA mediated-macrophage pyroptosis through acetylated MCU-dependent calcium uptake
Source: Cell Death Discov. 2025 Oct 7;11:449. doi: 10.1038/s41420-025-02746-9 (PMC12504438; doi:10.1038/s41420-025-02746-9)

Fig 1

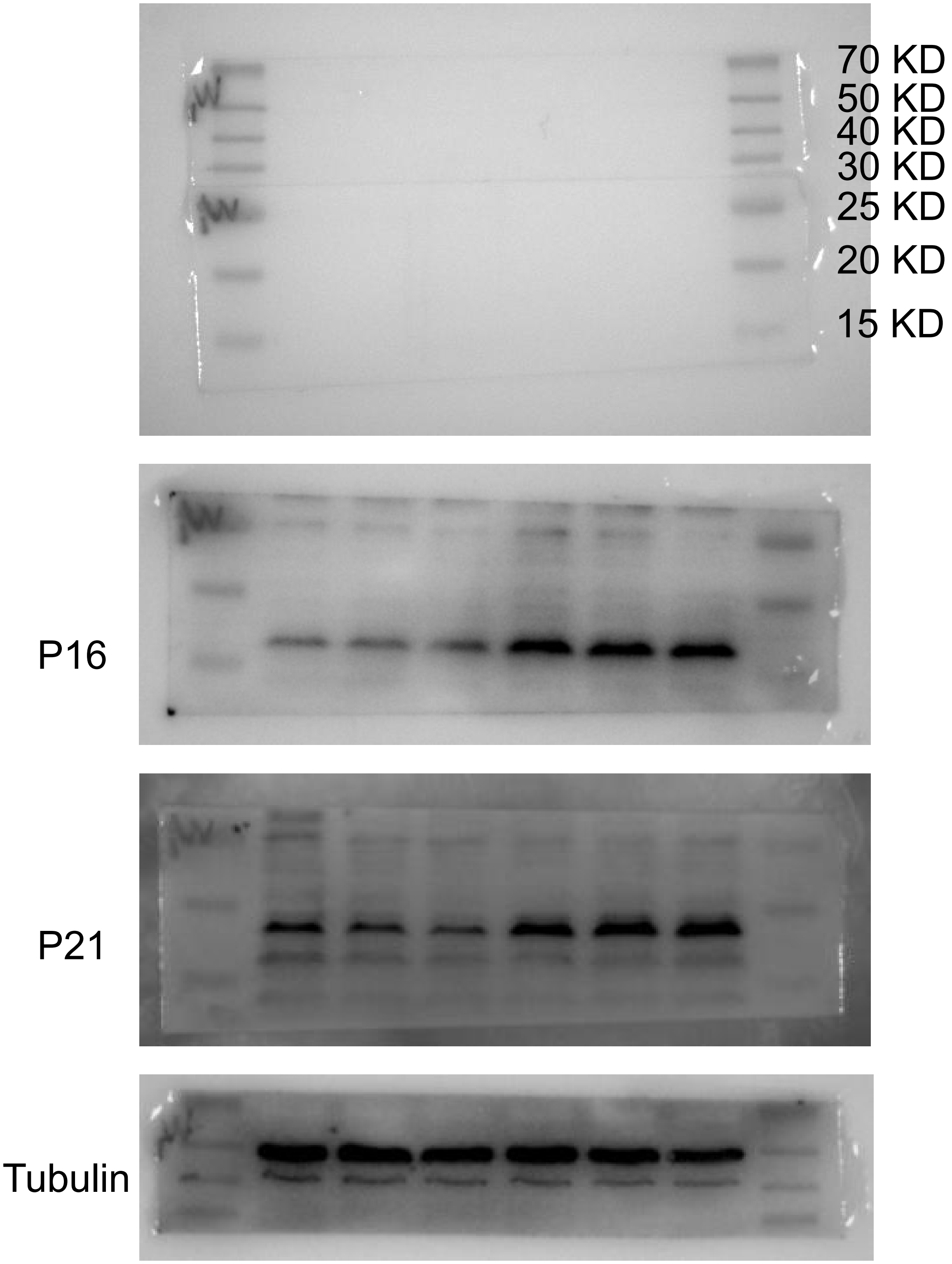

Fig 2

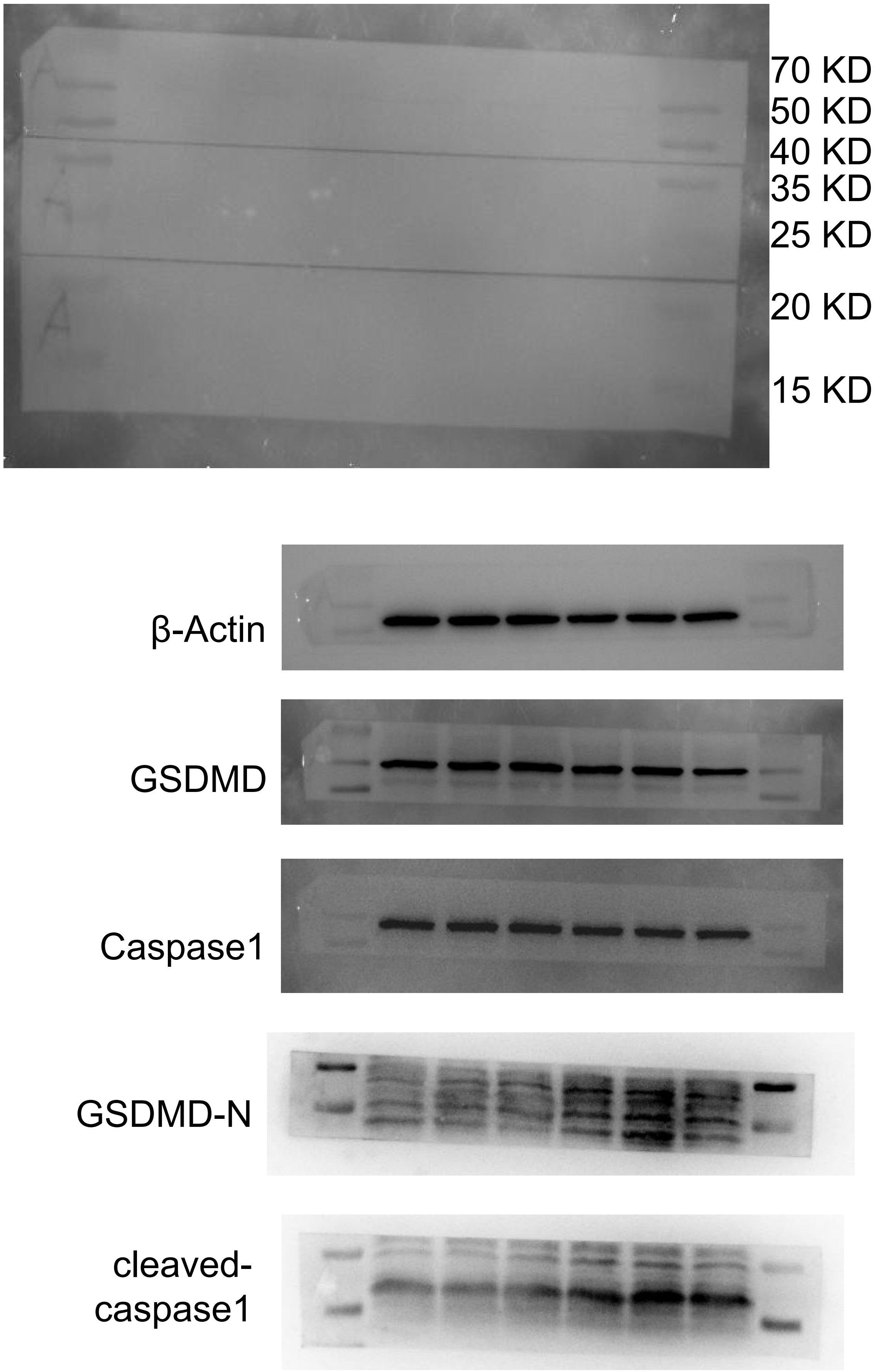
70 KD
50 KD
40 KD
35 KD
25 KD
20 KD
15 KD
β-Actin
GSDMD
Caspase1
GSDMD-N
cleaved-
caspase1

Fig 4

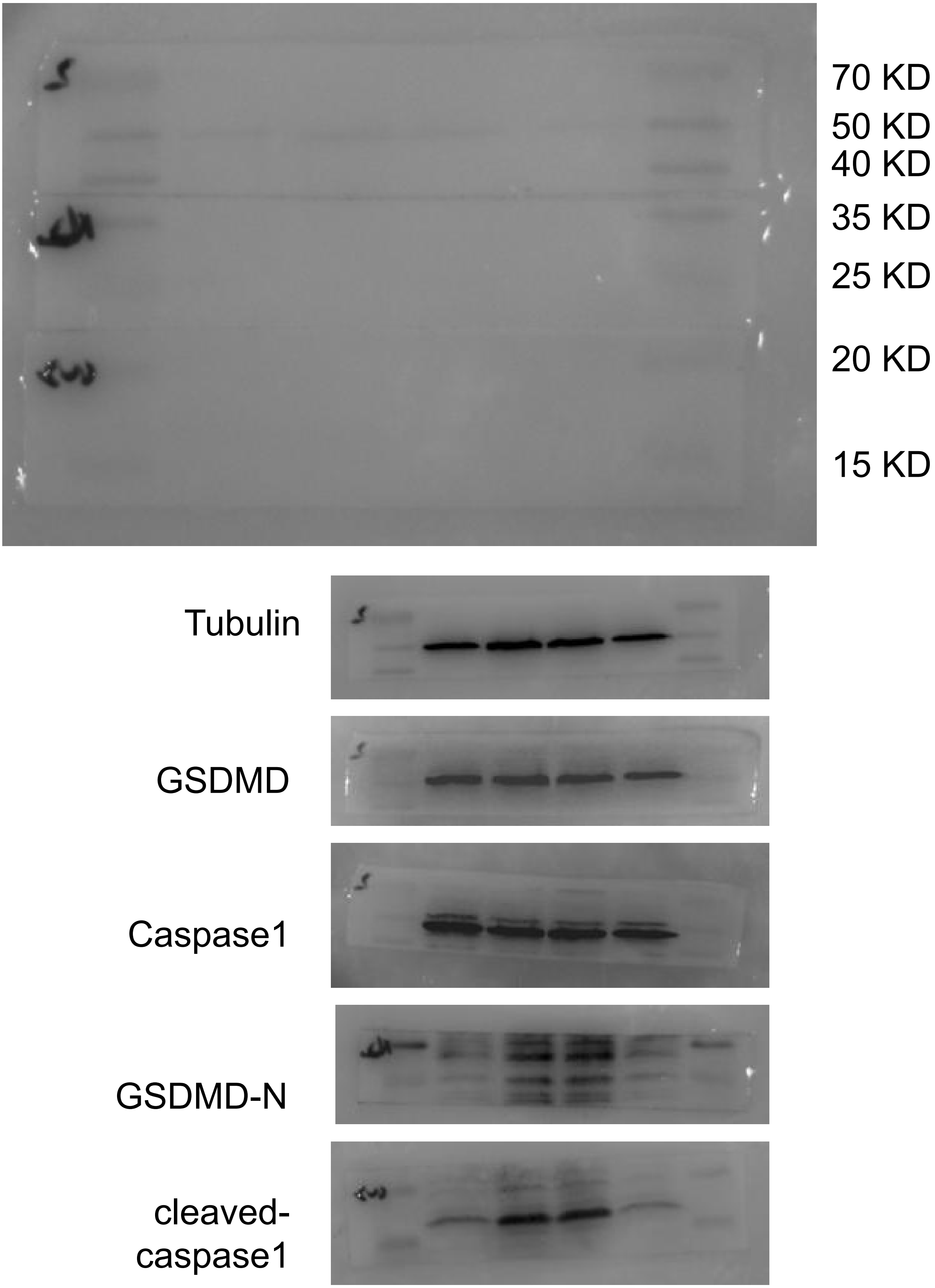

70 KD
50 KD
40 KD
35 KD
25 KD
20 KD
15 KD
Tubulin
GSDMD
Caspase1
GSDMD-N
cleaved-caspase1

Fig 6

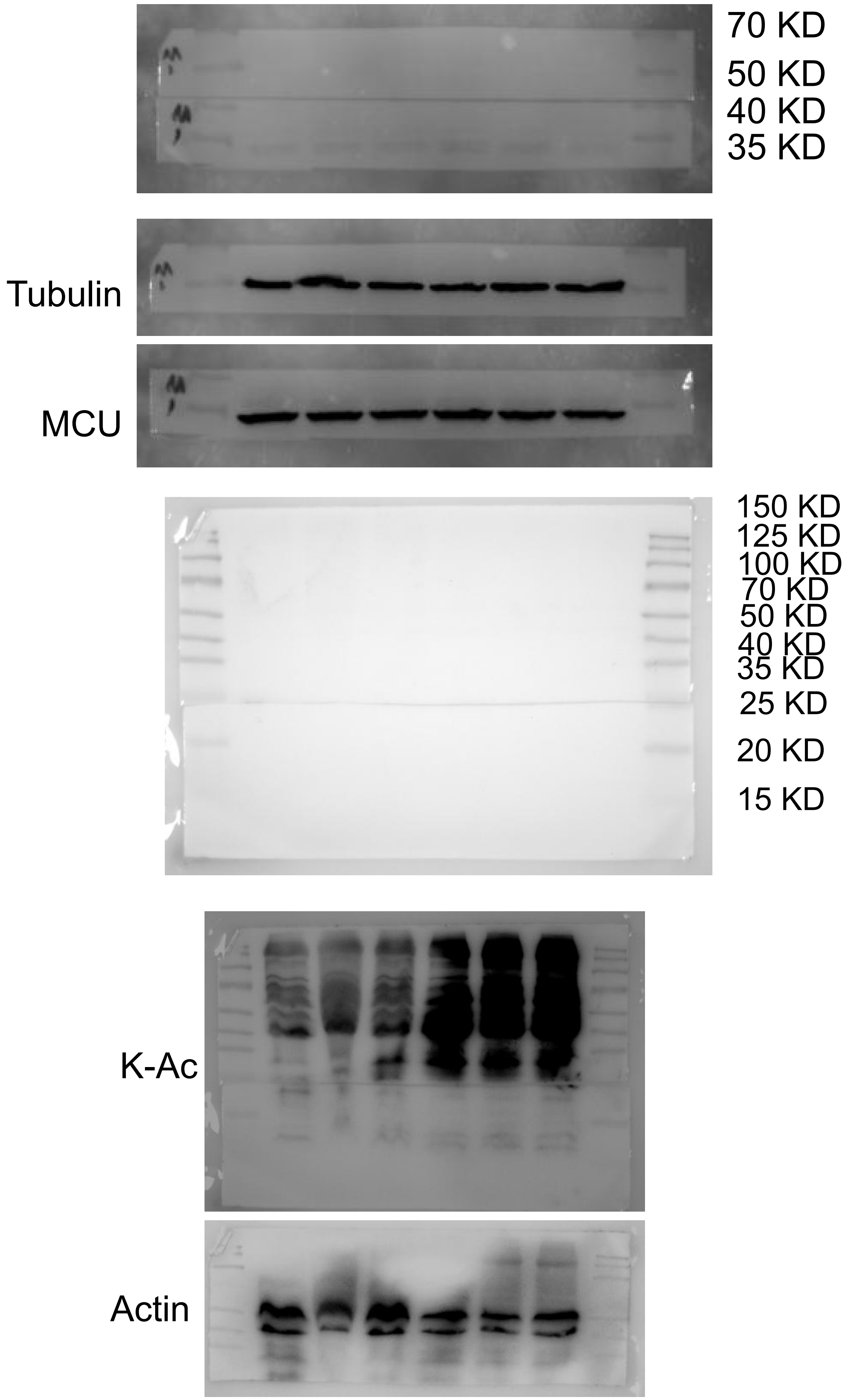
70 KD
50 KD
40 KD
35 KD
Tubulin
MCU
150 KD
125 KD
100 KD
70 KD
50 KD
40 KD
35 KD
25 KD
20 KD
15 KD
K-Ac
Actin

Fig 6

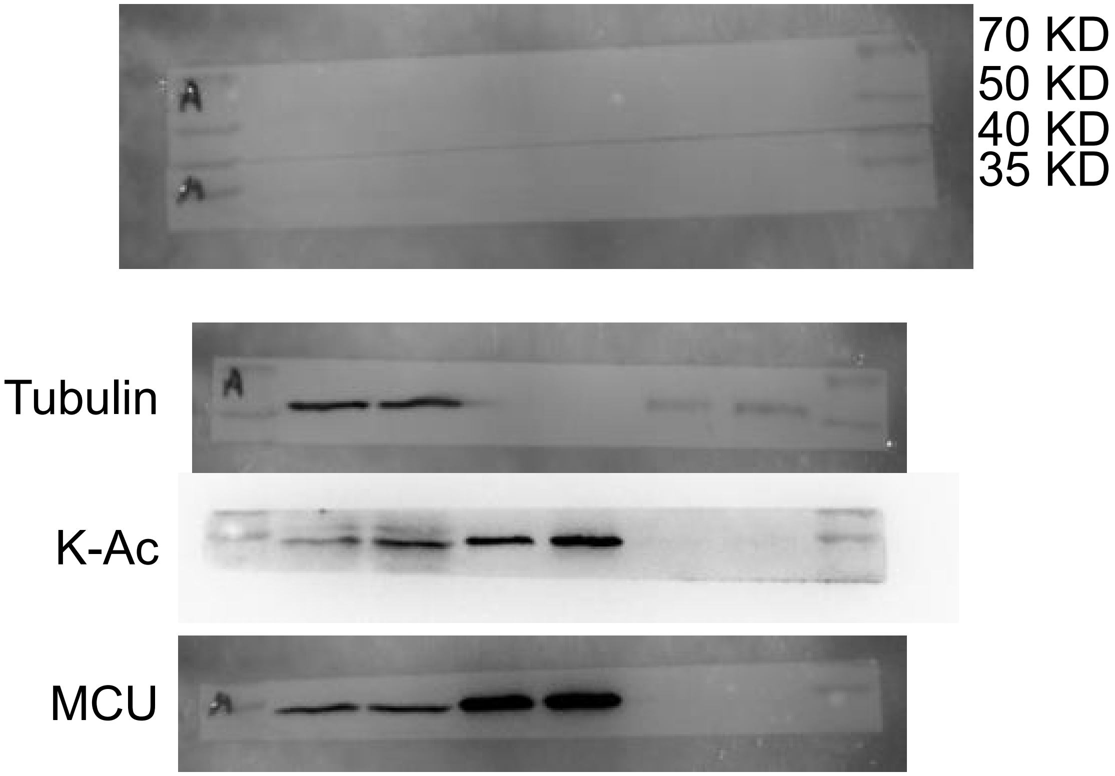

Fig 8

70 KD
50 KD
40 KD
35 KD
25 KD
20 KD
15 KD

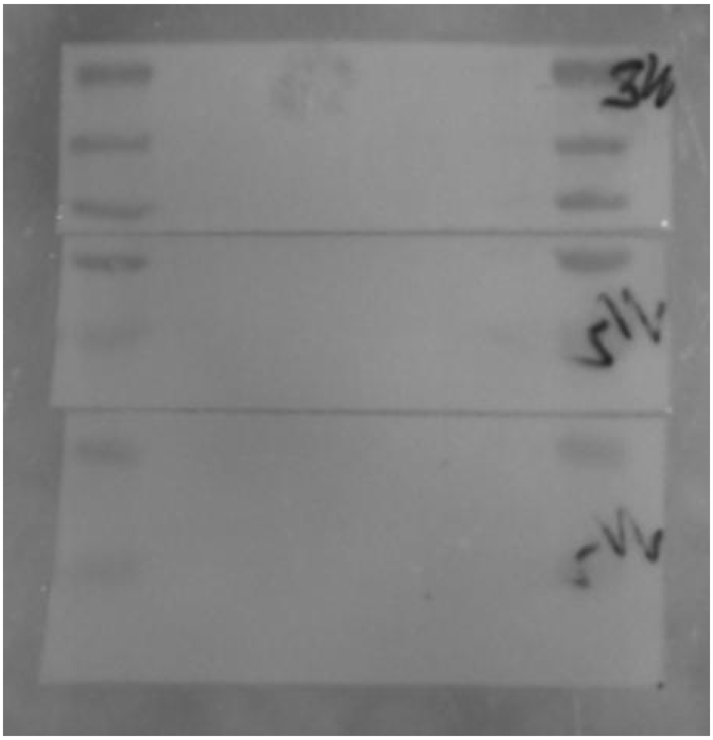

Caspase1

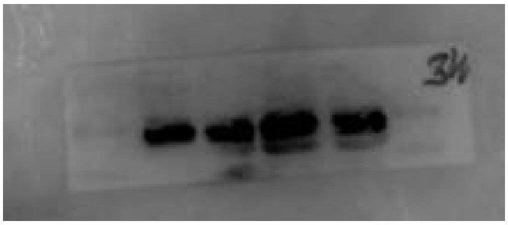

β-Actin

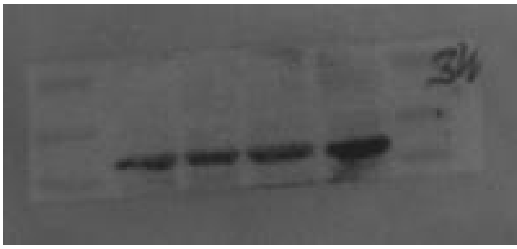

GSDMD

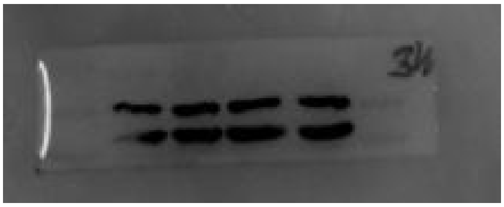

GSDMD-N

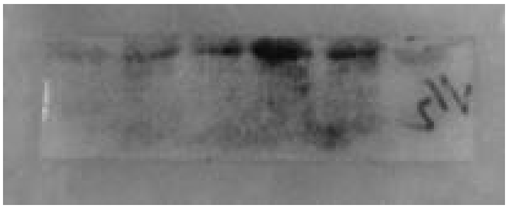

cleaved-caspase1

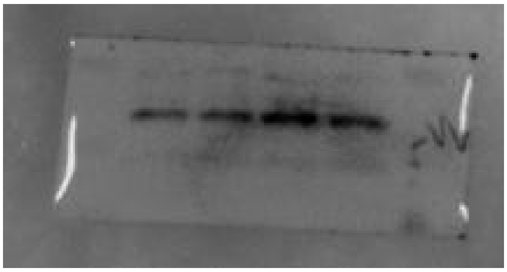

Fig S1

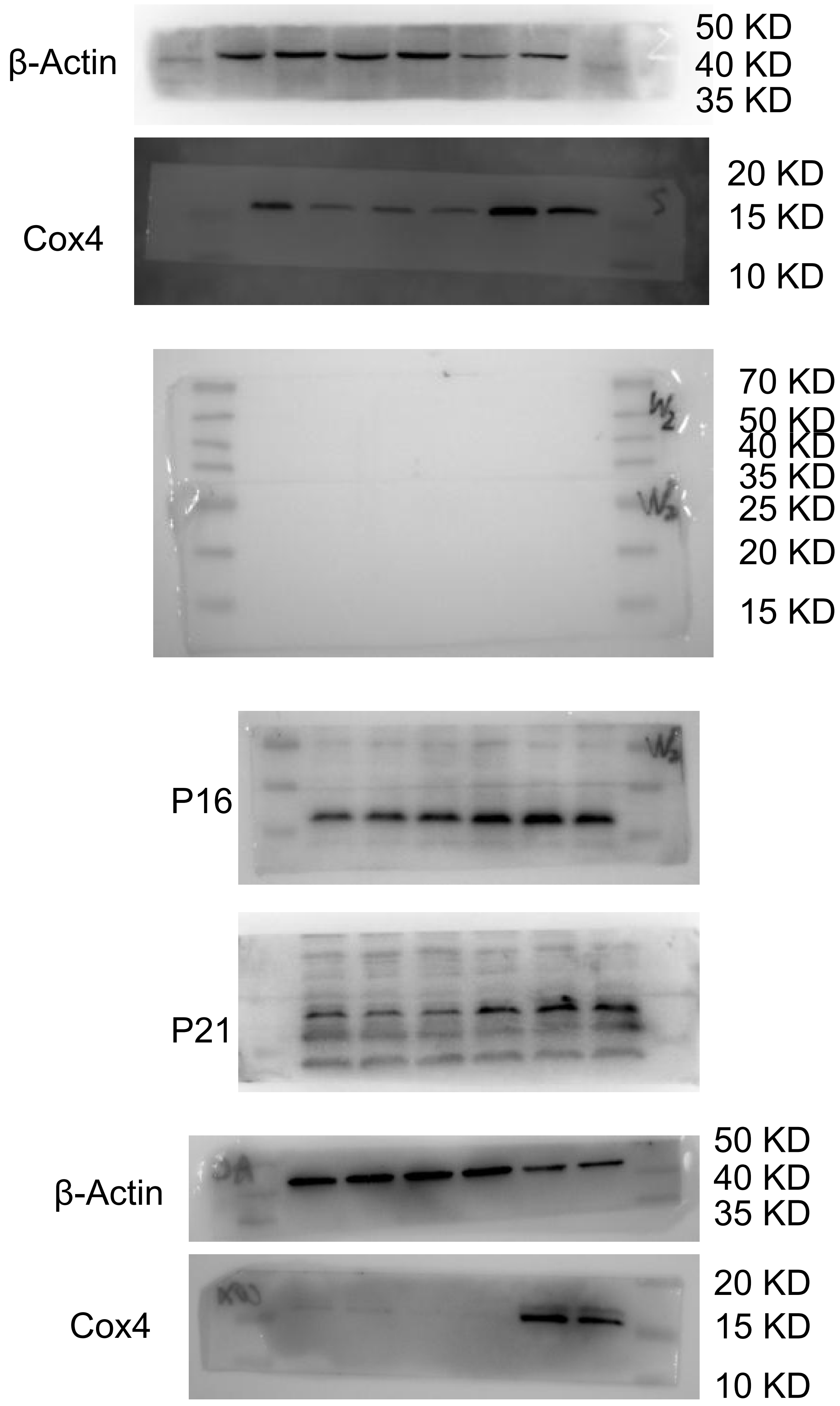
β-Actin
50 KD
40 KD
35 KD
Cox4
20 KD
15 KD
10 KD
70 KD
50 KD
40 KD
35 KD
25 KD
20 KD
15 KD
P16
P21
β-Actin
50 KD
40 KD
35 KD
Cox4
20 KD
15 KD
10 KD

Fig S3

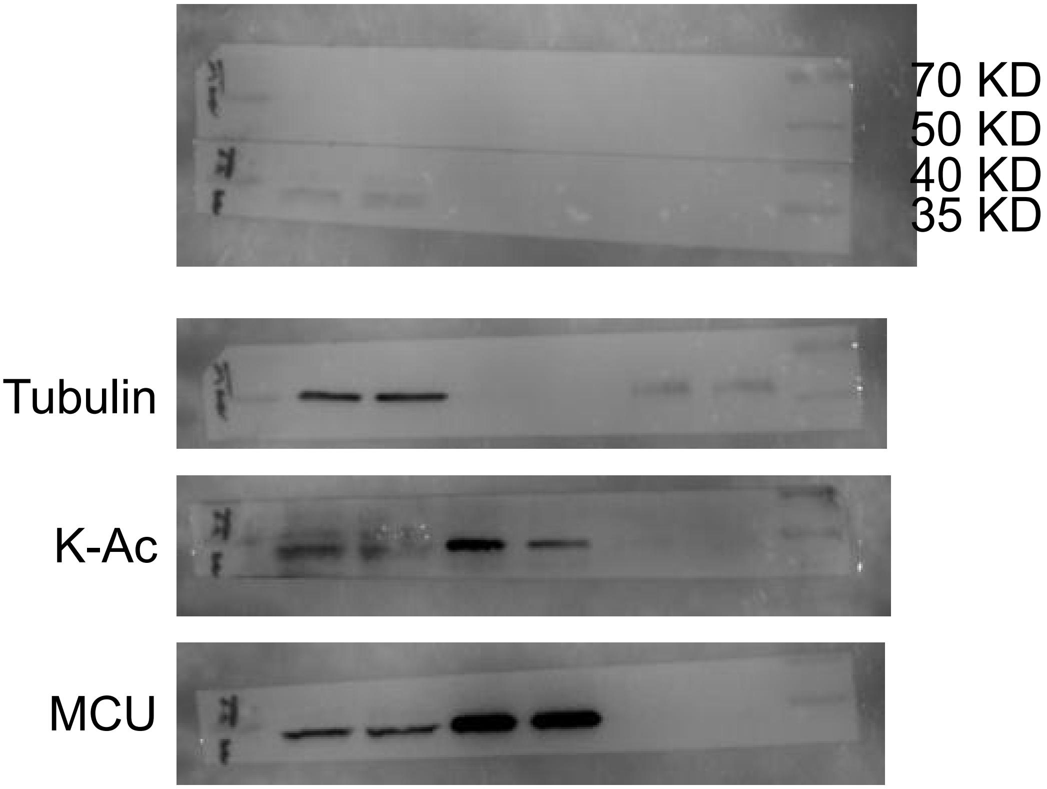

Supplement: Supplementary file 2 — Dataset 1 [file 41420_2025_2746_MOESM2_ESM.pdf]
